# Protective versus deleterious roles of pyroptosis in *Mycobacterium avium* and *Mycobacterium marinum* infections

**DOI:** 10.1038/s41419-026-08810-1

**Published:** 2026-04-30

**Authors:** Wanbin Hu, Federico Pagliaro, Herman P. Spaink, Annemarie H. Meijer

**Affiliations:** Institute of Biology Leiden, Leiden University, Einsteinweg 55, Leiden, The Netherlands

**Keywords:** Infection, Inflammation

## Abstract

*Mycobacterium avium*, a slow-growing nontuberculous mycobacterium (NTM), is the main cause of life-threatening NTM infections, which are globally on the rise. Unlike *Mycobacterium tuberculosis* and *Mycobacterium marinum*, *M. avium* lacks ESX-1, a subtype of Type VII secretion system (T7SS) and a key virulence determinant. The absence of ESX-1 in *M. avium* raises questions about its alternative intracellular survival strategies. To investigate *M. avium* pathogenesis, we exploited our recently established infection model in zebrafish larvae, enabling live imaging of early host-pathogen interactions. Macrophage depletion significantly increased *M. avium* burden and larval mortality, while neutrophil depletion had no major effect, emphasizing macrophages as key defenders against *M. avium*. In support, imaging of *tnfa* activation showed that macrophages polarized to a proinflammatory phenotype. However, like *M. marinum*, *M. avium* exploits chemokine receptor *Cxcr3.2* signaling in macrophages for its expansion in granuloma-like clusters. Both *M. avium and M. marinum* preferentially infected macrophages, but *M. avium*-induced granuloma-like clusters were more compact and exhibited less cell death. Supporting this, lytic cell death pathways were enriched in *M. marinum* but not *M. avium* transcriptome signatures. Consequently, we investigated pyroptosis, an important form of inflammation-induced lytic cell death. We found that knockdown of critical mediators of pyroptosis, namely inflammatory caspase a (*caspa*) and gasdermin Eb (*gsdmeb*), produced opposing effects on the two mycobacterial pathogens, indicating a host-protective role during *M. avium* infection, while exacerbating *M. marinum* growth. These findings highlight the interaction with host cell death signaling as a determining factor for the pathogenic potential of mycobacterial species.

## Introduction

Mycobacteria are the causative agents of pulmonary and extrapulmonary infections, which affect immunocompromised individuals in particular. While tuberculosis (TB), caused by *Mycobacterium tuberculosis*, remains a serious worldwide epidemic, nontuberculous mycobacteria (NTM) infections have recently gained global attention due to their rising incidence [1]. *M. avium* is responsible for the majority of NTM disease cases worldwide, which have a poor prognosis (27% mortality rate) due to *M. avium*’s intrinsic antibiotic resistance [2]. There is limited understanding of how *M. avium* causes pathogenesis in comparison with *M. tuberculosis* or its close relative *M. marinum*, often used as a surrogate model for tuberculosis. Notably, the virulence factor ESX-1, belonging to type VII secretion systems (T7SSs), is essential for immune evasion of both *M. tuberculosis* and *M. marinum* [3, 4]. However, ESX-1 is absent in *M. avium* [5]. which raises fundamental questions about how this pathogen establishes and propagates infection.

The roles of macrophages and neutrophils have been extensively studied in *M. tuberculosis* and *M. marinum* infections. Infected macrophages recruit additional macrophages and neutrophils, eventually leading to the formation of granulomas, inflammatory clusters that form the pathological hallmark of TB [6]. Macrophages and neutrophils recognize pathogen-associated molecular patterns of invading mycobacteria through pattern recognition receptors, which trigger the secretion of pro-inflammatory cytokines and defense mechanisms such as reactive oxygen production and antimicrobial autophagy [7, 8]. Upon eventual cell death, infected macrophages are engulfed by newly recruited macrophages in a process known as efferocytosis [8, 9]. Repeated cycles of infected macrophage death and phagocytosis by incoming macrophages promote infection spread and early granuloma formation [10, 11]. The ESX-1 secretion system plays a critical role in this process by promoting mycobacterial survival within macrophages and facilitating cell-to-cell spread [10]. In fact, ESX-1-mediated virulence has been identified as a main driver of granuloma formation due to its stimulatory effects on macrophage polarization and motility [10]. Although macrophages are the primary immune cells associated with mycobacterial infections, protective or deleterious roles for other phagocytic cells, including neutrophils, have also been identified, but are less well understood [9, 12]. It remains to be elucidated how the lack of ESX-1 in mycobacterial species such as *M. avium* impacts macrophage and neutrophil behavior and the mechanisms underlying granuloma formation.

Cell death plays a crucial role in shaping the host immune response to mycobacterial infections. Various forms of programmed cell death, including apoptosis, necroptosis, and pyroptosis, have been implicated in controlling or facilitating bacterial dissemination [13]. While apoptosis is generally considered a host-protective mechanism, necroptosis and pyroptosis represent lytic forms of cell death and are associated with the release of inflammatory signals [13]. In this context, pyroptosis is of particular interest because it connects inflammasome signaling, interleukin processing, and cell death. Triggering of pyroptosis by inflammasome activation leads to caspase-mediated cleavage and activation of interleukins (IL1/18) as well as gasdermin proteins [14, 15]. These results in the formation of gasdermin pores in the plasma membrane, mediating interleukin secretion and eventually cell lysis. It has been suggested that *M. tuberculosis* exploits lytic cell death pathways, including pyroptosis, to escape from host cells and enhance its spreading [13, 15]. Similarly, both pyroptotic and necroptotic cell death processes have been shown to exacerbate *M. marinum* growth in zebrafish larvae [16, 17]. However, the roles of cell death programs in *M. avium* infection have not been explored.

Understanding how different mycobacterial species interact with host immune cells is critical for elucidating mechanisms of pathogenesis. To investigate this, we focused on the roles of macrophages and neutrophils during *M. avium* infection. We used the zebrafish (*Danio rerio*) model, which offers distinct advantages for studying mycobacterial infections in vivo: its optical transparency at the larval stages allows real-time imaging of immune responses, its genetic tractability enables targeted manipulation of host pathways, and its innate immune system shares key functional similarities with that of mammals [18, 19]. Zebrafish larvae have been extensively used to model *M. marinum* infection, which contributed important insights into the host-pathogen interaction and granuloma formation that can be extrapolated to *M. tuberculosis* pathogenesis [6, 20–22]. Recently, we have established an *M. avium* infection model in zebrafish larvae, providing a valuable tool to explore the pathogenesis of *M. avium* infection in vivo in the same host model as *M. marinum* [23]. Using both systemic and localized infection approaches combined with confocal imaging and macrophage depletion, we demonstrate here that macrophages are indispensable for controlling *M. avium*. While many of the responses of macrophages and neutrophils were similar between *M. avium* and *M. marinum* infection, transcriptomic analysis indicated differential regulation of cell death pathways between these two pathogens, prompting us to further examine the role of lytic cell death during *M. avium* infection. Through genetic knockdowns targeting key pyroptosis mediators, we found that pyroptosis, unlike in *M. marinum* infection, plays a protective role during *M. avium* infection. Overall, our findings reveal that, despite lacking the major virulence factor ESX-1, *M. avium* shares several pathological hallmarks with *M. marinum* but differs markedly in its ability to induce and exploit host cell death.

## Results

### *M. avium* expands through granuloma-like aggregates in both systemic and localized infections

**Fig. 1:**
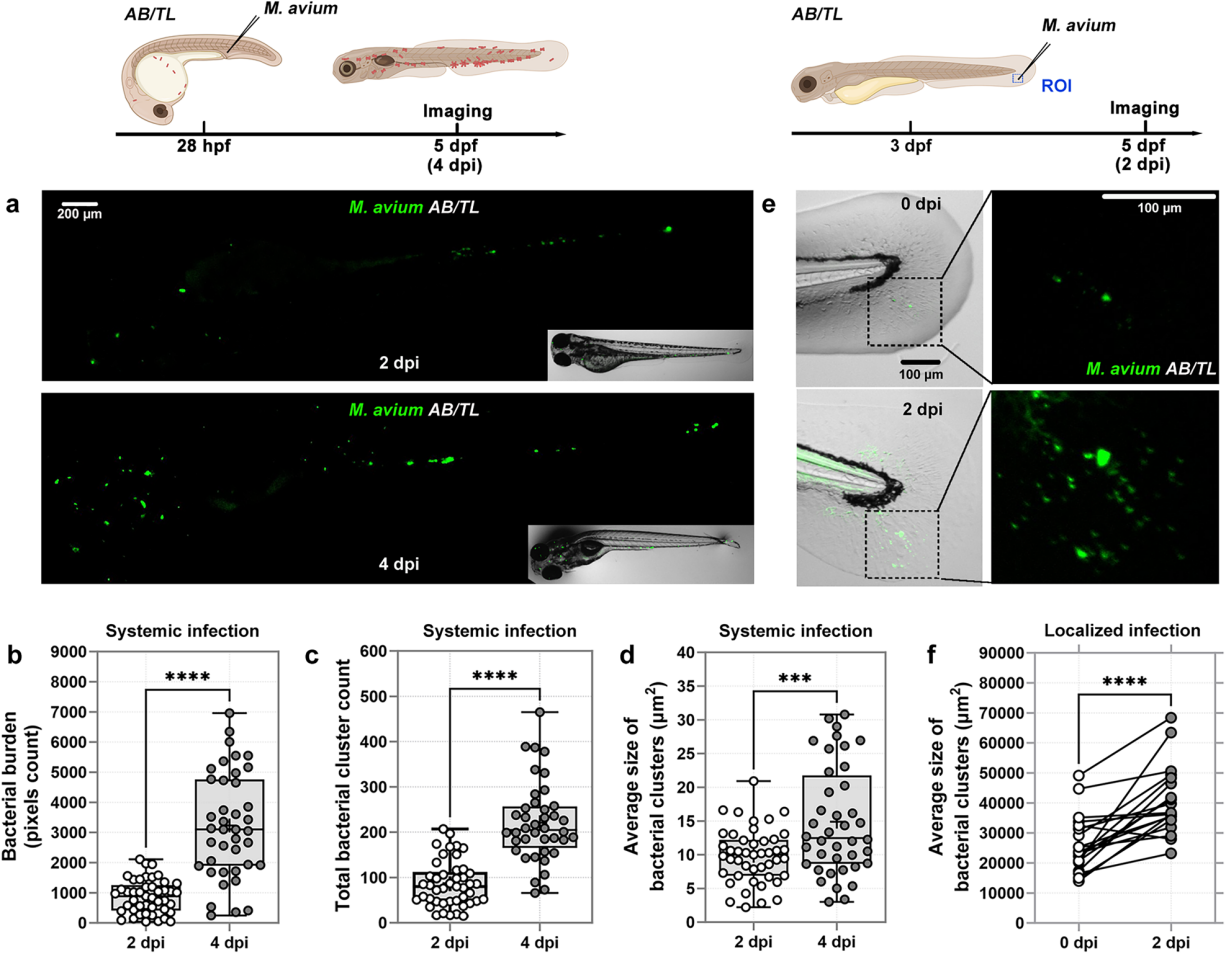
Granuloma-like clusters formation upon a systemic and a localized *M. avium* infection. **a**–**d** zebrafish embryos (genotype *AB/TL*) were systematically infected at 28 hpf with mWasabi-labeled *M. avium* strain MAC 101 at a dose of 4500 CFU by caudal vein infection. Data are based on two independent experiments (*n* = 45 at 2 dpi, *n* = 40 at 4 dpi, *N* = 2). **a** Representative images of systemically *M. avium*-infected larvae at 2 dpi (top panel) and 4 dpi (bottom panel). Scale bar: 200 µm. **b** Bacterial burden quantification of zebrafish larvae upon *M. avium* systemically infection. **c** Quantification of total bacterial cluster numbers in *M. avium* systemically infected zebrafish larvae. **d** Quantification of the average area of *M. avium* clusters. **e**, **f** embryos were locally infected at 3 dpf with mWasabi-labeled *M. avium* strain MAC 101 at a dose of 50 CFU by tail fin infection (*n* = 19, *N* = 2). **e** Representative images of the tail fin regions of locally *M. avium*-infected larvae at 0 dpi (top panel) and 2 dpi (bottom panel). Scale bar: 100 µm. **f** Increase in the average size of *M. avium* infection clusters in the tail fins of individual larvae between 0 and 2 dpi (*n* = 19, *N* = 2). Statistical significance of difference was determined by an unpaired (**b**–**d**) or paired (**f**) *t*-test. *** *P* < 0.001, **** *P* < 0.0001.

Our previous study established a systemic *M. avium* infection model using intravenous injection [23]. We first applied this systemic infection by injecting mWasabi-labeled *M. avium* into the blood island of wild-type *AB/TL* zebrafish at 28 h post-fertilization (hpf) (Fig. 1a–d). Consistent with our previous findings [23], granuloma-like infection foci were visible at 4 days post-infection (dpi) and bacterial burden was significantly increased between 2 and 4 dpi (Fig. 1a, b). Additionally, both the total number of bacterial clusters (Fig. 1c) and the average area of individual bacterial clusters (Fig. 1d) increased over time. Similarly, the formation of granuloma-like clusters of infected cells was observed following localized tail fin infection at 3 day-post fertilization (dpf) (Fig. 1e). Quantification of bacterial cluster size in the tail fin region further confirmed a significant increase between 0 and 2 dpi (Fig. 1f). These findings demonstrate that both systemic and localized infections in zebrafish larvae promote *M. avium* expansion, accompanied by the formation of granuloma-like clusters similar to those previously observed in *M. marinum* infection, providing comparable host models to study macrophage and neutrophil functions in defense against these mycobacterial pathogens (Fig. S1).

### *M. avium*-infected neutrophils are engulfed by macrophages or undergo reverse migration

**Fig. 2:**
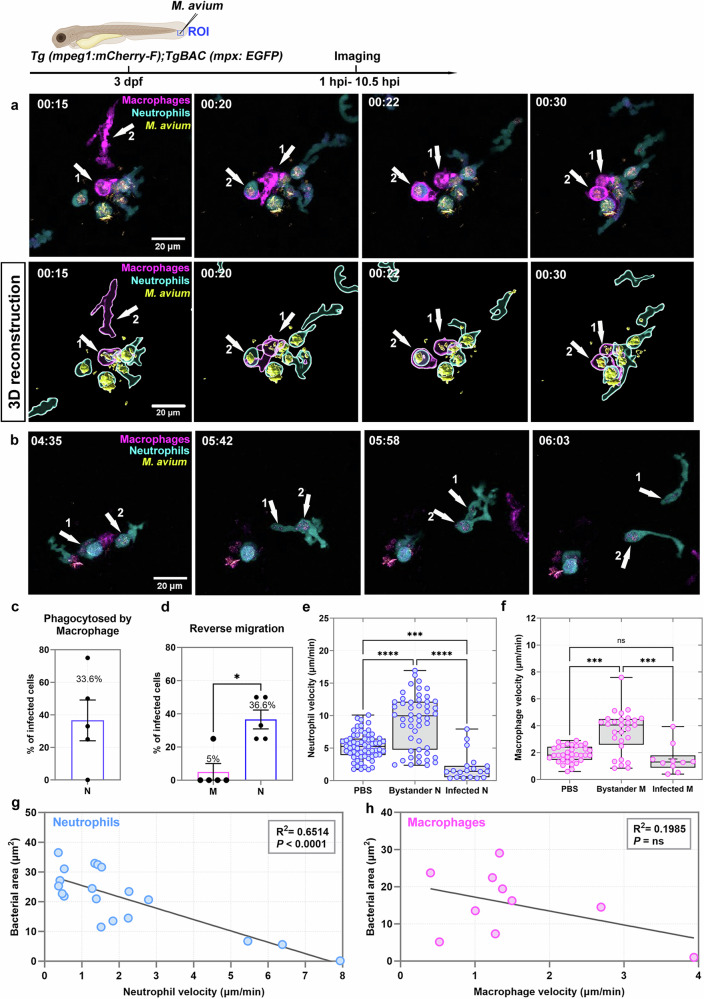
Quantification of macrophage and neutrophil behaviors in *M. avium*-infected zebrafish larvae. *Tg(mpeg1:mCherry-F); TgBAC(mpx:EGFP)* embryos were locally infected at 3 dpf with E2Crimson-labeled *M. avium* strain MAC 101 at a dose of 50 CFU by tail fin infection. Live imaging was taken from 1 to 10.5 hpi with a time interval of 1 min. Five *M. avium*-infected larvae and five PBS-infected larvae were observed over time (*N* = 5). **a** Representative images showing macrophages engulfing infected neutrophils. White arrows show macrophages phagocyting *M. avium*-infected neutrophils. The lower panel shows 3D reconstruction images of the upper panel. Scale bar: 20 µm. See Movie S1 for more details of panel (**a**), see Fig. S3 for the other example. **b** Representative images of neutrophils performing reverse migration. White arrows show the reverse-migrated *M. avium*-infected neutrophils. See Movie S2 for more details of panel (**b**). **c** Percentage of infected neutrophils (N) engulfed by macrophages (M). The total number of infected neutrophils (engulfed and non-engulfed) analyzed was 29. **d** Percentage of infected cells with reverse migration behavior. The total number of infected macrophages and infected neutrophils analyzed was 15 and 29, respectively. **e**, **f** migration velocity comparison between neutrophils (**e**) and macrophages (**f**) in PBS-injected larvae and *M. avium*-infected larvae. “Bystander N or M” or “Infected N or M” refers to respectively non-infected and infected cells in the infected zebrafish larvae. See Movie S3 and Movie S4 for more details about cell tracking of macrophages and neutrophils in *M. avium*-infected larvae. **g**, **h** correlation analysis between bacteria area and infected neutrophils (**g**) or macrophages (**h**). In (**c**, **d**), data are shown in mean ± SEM, each dot represents one larva. In (**d**), statistical significance was determined using two-tailed Mann–Whitney *U*-tests. In (**e**, **f**), Kruskal–Wallis one-way analysis followed by Dunn’s multiple comparison test was used to assess statistical significance. ns, not significant, *P < 0.05, ***P < 0.001, ****P < 0.0001.

To determine which leukocyte type *M. avium* preferentially infects, we quantified bacterial residence within macrophages and neutrophils using the double transgenic line *Tg(mpeg1:mCherry-F);TgBAC(mpx:EGFP)* (Fig. S2). Based on the described tissue-dependency of the phagocytic properties of macrophages and neutrophils [24]. We expected that localized tail fin injection would result in a higher level of neutrophil residence, while systemic injection into the blood island would lead to higher macrophage residence. While quantification confirmed this general pattern, we found that the *M. avium* predominantly resides within macrophages rather than neutrophils in both the localized (59.26%–62.36% from 1–9 hpi) and systemic (more than 90% at 4 hpi) infection models (Fig. S2). To better understand the roles of macrophages and neutrophils in defense against *M. avium*, we next analyzed the dynamic behaviors of these infected cells using live imaging on zebrafish larvae infected via the tail fin, starting from 1 h post-infection (hpi) (Fig. 2). This revealed that infected neutrophils were engulfed by newly arriving macrophages (Fig. 2a and Movie S1). In addition, we observed reverse migration of infected neutrophils away from the primary infection site (Fig. 2b and Movie S2). While we frequently observed macrophages engulfing infected neutrophils, phagocytosis of infected macrophages was not detected within the same time frame (Figs. 2a and Fig. S3; Movie S1). Quantification revealed that 33.6% of infected neutrophils were phagocytosed by macrophages, whereas infected neutrophils were not engulfed by other neutrophils (Fig. 2c). Our time-lapse analysis further shows that before undergoing cell death, infected neutrophils can contain *M. avium* for a sufficient amount of time to be engulfed by macrophages and undergo reverse migration (Fig. 2b and Movie S2). Notably, a higher percentage of infected neutrophils (36.6 ± 5.67%) exhibited reverse migration behavior compared to infected macrophages (5 ± 5%) (Fig. 2d). Bystander neutrophils and macrophages migrated faster than their infected counterparts (Fig. 2e, f; Movie S3 and Movie S4). Additionally, a negative correlation was observed between the migration speed of neutrophils and their bacterial area, whereas macrophage migration speed remained unaffected by the infected area (Fig. 2g, h). Together, these results show that macrophages are effective in engulfing infected neutrophils, while neutrophils display more frequent reverse migration that could facilitate *M. avium* dissemination.

### Macrophages display proinflammatory polarization during *M. avium* infection

**Fig. 3:**
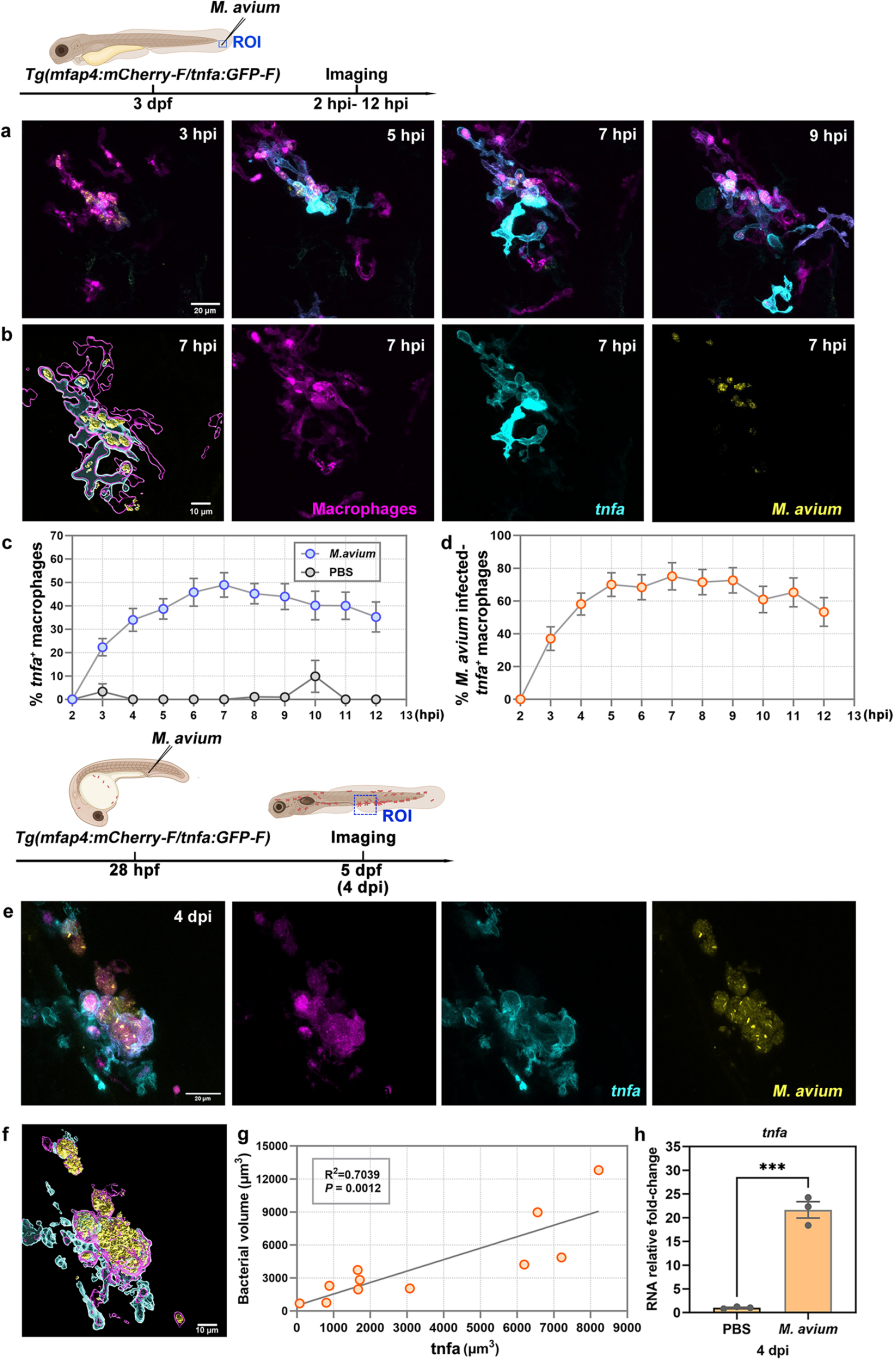
Macrophage polarization in *M. avium*-infected zebrafish larvae. **a**–**d** *Tg(mfap4: mCherry- F/tnfa: GFP- F)* larvae were injected with PBS or ~50 CFU *M. avium* E2Crimson in the tail fin. **a** Representative confocal image showing *tnfa* and macrophage (*mfap4*) reporter gene expression in the tail fin region after *M. avium* infection at 3 dpf. Scale bar: 20 µm. **b** 3D reconstruction of *M. avium*-infected *Tg(mfap4: mCherry- F/tnfa: GFP- F)* larva at 7 hpi. Scale bar in the 3D reconstruction image: 10 µm. **c** Quantification of the percentage of *tnfa*^*+*^ macrophages from 2 hpi to 12 hpi. **d** Quantification of the percentage of *M. avium*-infected macrophages that were positive for *tnfa* from 2 hpi to 12 hpi. **e** Representative confocal images showing the *tnfa* expression in the caudal hematopoietic tissue (CHT) of *M. avium* infected larva at 4 dpi. Scale bar: 20 µm. **f** 3D reconstruction image of panel (**e**). Scale bar: 10 µm. **g** Volume of *tnfa* expressed to granuloma-like clusters as a function of the granuloma-like clusters volume at 4 dpi. **h** The expression level of *tnfa* was determined at 4 dpi by qPCR. In (**c**, **d**), data (mean ± SEM) were combined from two independent experiments with 15 fish in total (*n* = 15, *N* = 2). In (**h**), data were derived from three biological replicates (*n* = 30 larvae per group, *N* = 3) and expressed relative to their corresponding mock injection (PBS) control, which is set at 1. Statistical significance of differences was determined by an unpaired *t*-test. *** *P* < 0.001.

To investigate whether macrophages that phagocytose *M. avium* adopt a pro-inflammatory phenotype, we investigated *tnfa* expression as a marker of M1-like proinflammatory polarization [25]. Time course images and 3D reconstruction revealed that the expression of *tnfa* was induced in *M. avium-*infected macrophages (Fig. 3a, b). Up to around 50% of the total number of macrophages were positive for *tnfa* in the *M. avium* infection group, while *tnfa* activation in the PBS-injected control group was negligible (Fig. 3c). Moreover, the peak level of infected macrophages with *tnfa* expression reached around 70% (Fig. 3d). The *tnfa* reporter expression in infected macrophages persisted at least until 4 dpi during systemic infection (Fig. 3e, f). Furthermore, a positive correlation was observed between the *M. avium* volume and *tnfa* expression volume (Fig. 3g). The findings with the *tnfa* reporter line were validated by qRT-PCR, which confirmed significantly higher *tnfa* expression in the *M. avium*-infected larvae compared to uninfected controls (Fig. 3h). These results demonstrate that *M. avium* infection induces a strong *tnfa* expression in zebrafish at both early and later infectious stages, as observed in our localized and systemic infection models.

### Macrophage ablation has a stronger effect on *M. avium* infection than neutrophil ablation

**Fig. 4:**
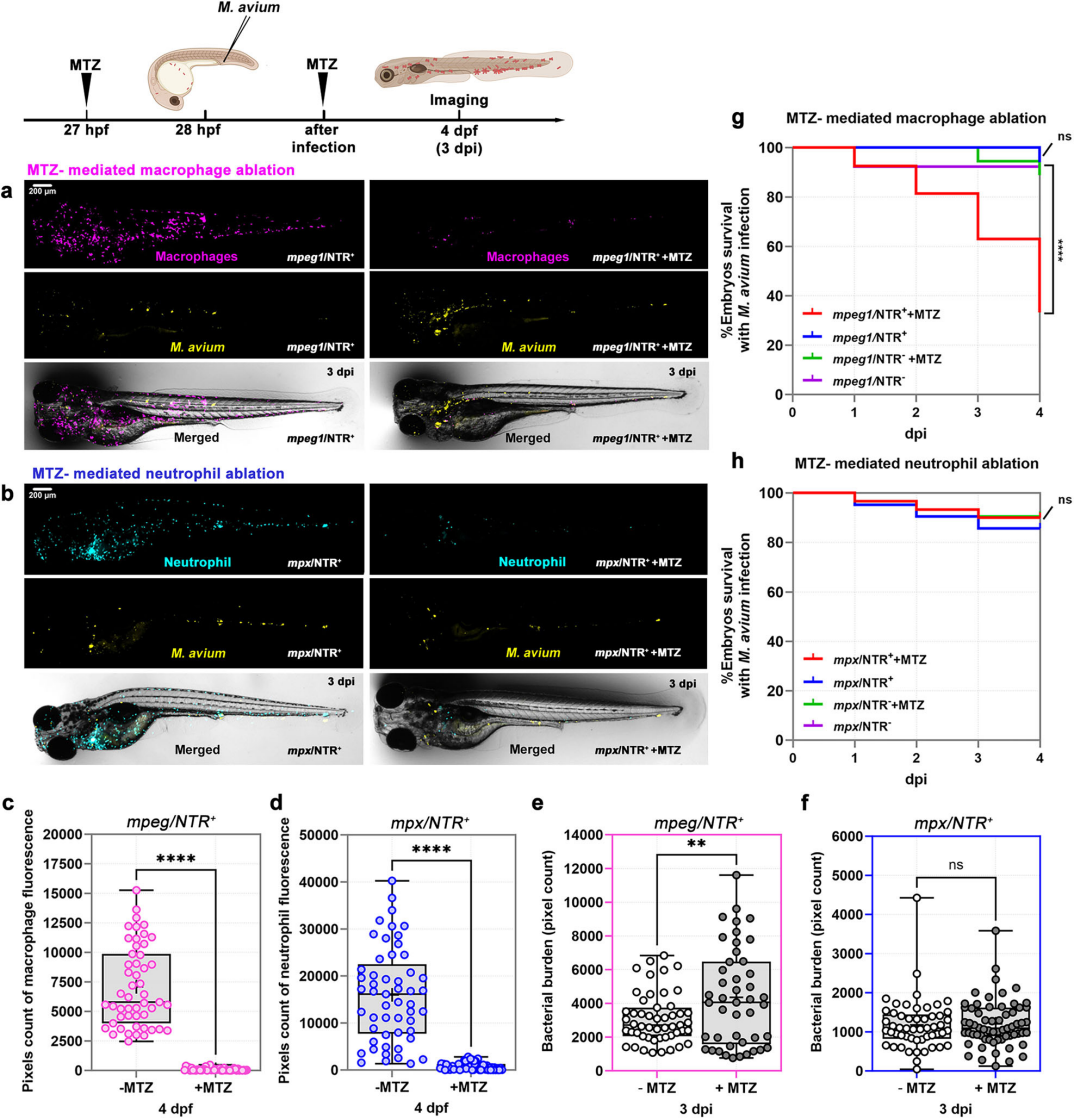
Effects of macrophage and neutrophil ablation on *M. avium* infection. *Mpeg1:NTR*^*+*^ and *Mpx:NTR*^*+*^ embryos were incubated with 5 mM MTZ at 27 hpf for 1 h. Subsequently, the MTZ-treated or control embryos were injected with ~4500 CFU E2Crimson *M. avium*. The images were taken at 3 dpi. **a**, **b** Representative images of MTZ-mediated macrophage ablation (**a**) and MTZ-mediated neutrophil ablation (**b**). Scale bar: 200 µm. **c**, **d** Quantification of total NTR^+^ macrophages pixel counts (**c**) or NTR^+^ neutrophils pixel counts (**d**) in control groups (−MTZ) and MTZ treatment groups ( + MTZ) at 4 dpf. **e**, **f** Pixel count of bacterial burden at 3 dpi in the control groups and MTZ-treated groups upon *M. avium* infection. In (**c**–**f**), data were combined from two independent experiments (*n* = 51, 45 in panels (**c**, **e**); *n* = 53, 63 in panels (**d**) and (**f**), *N* = 2). Statistical significance of difference was determined by using two-tailed Mann–Whitney *U*-tests. ns, not significant, ** *P* < 0.01, **** *P* < 0.0001. **g**, **h** Percentage of surviving larvae after *M. avium* infection in the absence of macrophages (**g**) or neutrophils (**h**). Data were combined with two replicates (*n* = 27 per condition in panel **g**, *n* = 30 per condition in panel **h**, *N* = 2), and the Log rank test was used. ns, not significant ****P < 0.0001. Figure S4 for the survival rate of the other control groups.

Given the proinflammatory polarization of macrophages, we hypothesized that they are essential for the containment of *M. avium*. To address this, we utilized the MTZ /nitroreductase (NTR) system, a well-established method for chemically inducing the ablation of macrophages or neutrophils in zebrafish larvae [26, 27]. MTZ treatment properly depleted the majority of macrophages or neutrophils in NTR^+^ zebrafish lines (Fig. 4a–d). Ablation of macrophages significantly increased the *M. avium* burden at 3 dpi (Fig. 4e), whereas no significant difference was observed after neutrophil ablation (Fig. 4f). Additionally, macrophage ablation resulted in over 60% larval mortality after *M. avium* infection at 4 dpi, while control groups exhibited more than 90% survival (Fig. 4g and Fig. S4). In contrast, neutrophil ablation had no significant impact on survival (Fig. 4h and Fig. S4). Our findings suggest that macrophages, but not neutrophils, are crucial for controlling *M. avium* infection in zebrafish larvae.

To further validate the protective function of macrophages, we employed liposome clodronate (Lipo-C)-mediated depletion [28]. Unlike the MTZ-based ablation system, which eliminates all NTR-expressing macrophages, Lipo-C specifically targets circulating monocytes/macrophages, leaving infected tissue-resident macrophages unaffected. This approach allowed us to distinguish the role of newly recruited macrophages from that of *M. avium*-infected cells in established granuloma-like aggregates. Following macrophage depletion with Lipo-C, we observed a marked reduction of macrophage fluorescence compared to PBS liposome (Lipo-PBS)-treated controls, confirming efficient ablation (Fig. S5a, b, d, e). When macrophages were depleted either at an early infection stage at 1 dpi or at a later stage at 3 dpi, bacterial burden significantly increased in the Lipo-C group compared to Lipo-PBS controls (Fig. S5a, c, d, f). These results corroborate the findings from the MTZ-mediated depletion experiments, demonstrating that macrophages are indispensable for controlling *M. avium* infection. Notably, because circulating macrophages were selectively ablated in this approach, these data also imply that newly recruited macrophages are critical for bacterial containment.

### *M. avium* exploits chemokine receptor signaling in macrophages

**Fig. 5:**
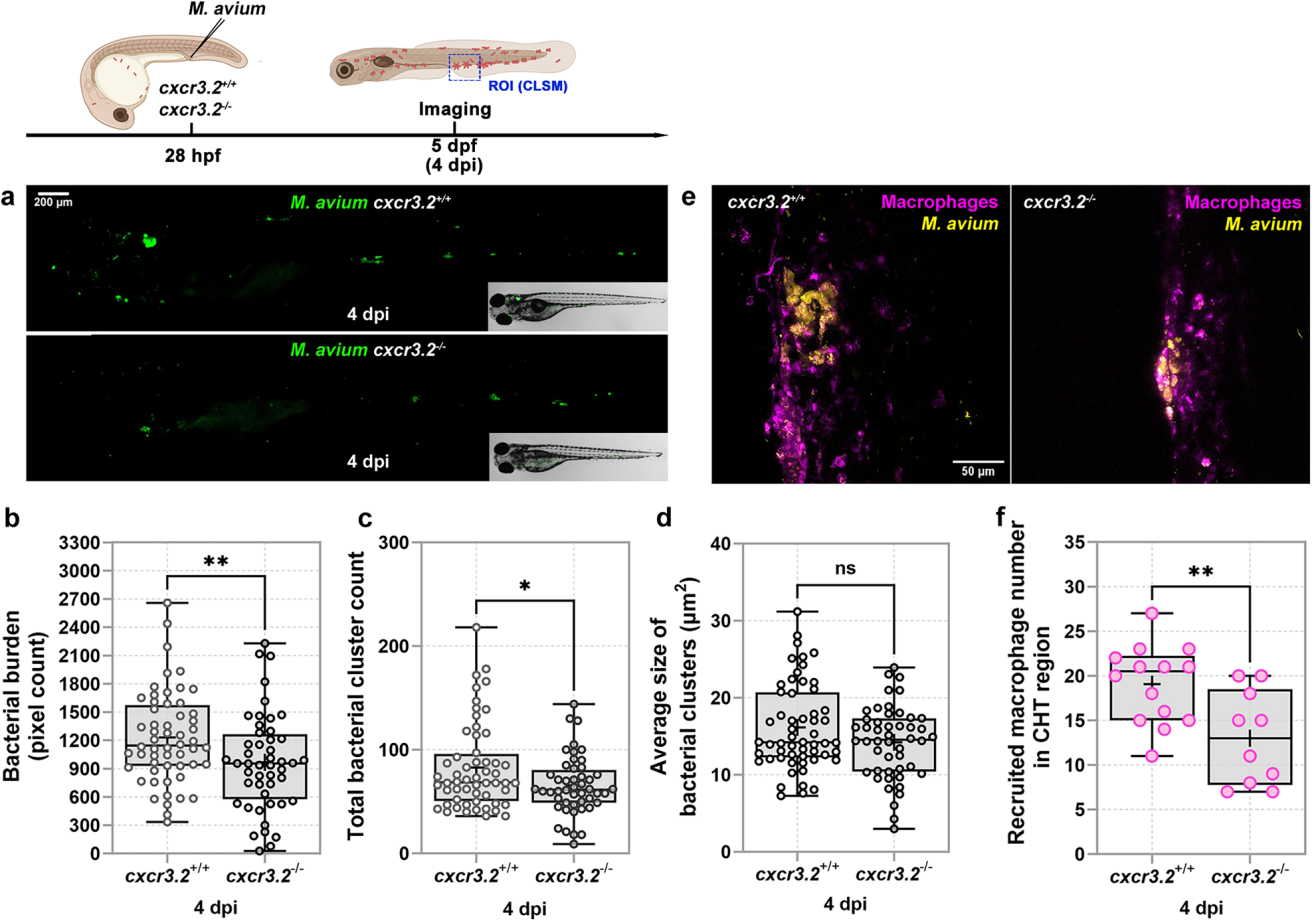
Effect of *cxcr3.2* mutation on macrophage behavior during *M. avium* infection. *Cxcr3.2*^*+/+*^ *and cxcr3.2*^*- /-*^ embryos were systemically infected at 28 hpf with mWasabi-labeled or E2Crimson-labeled *M. avium* strain MAC 101 at a dose of 4500 CFU by caudal vein infection. **a** Representative images of *M. avium*-infected *cxcr3.2*^*+/+*^ *and cxcr3.2*^*-/-*^ larvae at 4 dpi. Scale bar: 200 µm. **b** Bacterial burden quantification of *cxcr3.2* zebrafish larvae upon *M. avium* infection. **c** Quantification of total bacterial cluster numbers in *M. avium* infected *cxcr3.2* zebrafish larvae. **d** Quantification of the average area of *M. avium* clusters in *M. avium*-infected *cxcr3.2* zebrafish larvae. In (**b**–**d**), data were from three independent experiments (*n* = 56, 48, *N* = 3). **e** Representative images of granuloma-like clusters in infected *cxcr3.2*^*+/+*^ *Tg* (*mpeg1:mCherry- F) and cxcr3.2*^*- /-*^ *Tg* (*mpeg1:mCherry- F)* larvae. Scale bar: 50 µm. **f** Quantification of recruited macrophage numbers in *M. avium*-infected *cxcr3.2* larvae at 4 dpi (*n* = 14, 10, *N* = 2). Statistical significance of the difference was determined by an unpaired *t*-test. ns, not significant, *P < 0.05, **P < 0.01.

In *M. marinum* infection, macrophages not only exert a protective effect but also facilitate the dissemination of infection [10, 20, 29]. This behavior of infected macrophages is determined largely by the ESX-1 virulence system of *M. marinum* [10, 30]. The lack of ESX-1 in *M. avium,* therefore, raised the question of whether macrophages are solely host-protective in the infection or might facilitate dissemination in an ESX-1-independent manner. We have previously identified Cxcr3 chemotactic signaling as a host pathway contributing to macrophage recruitment and bacterial dissemination in *M. marinum*-infected zebrafish larvae [20, 31]. Additionally, our previous transcriptome analysis revealed a significant upregulation of *cxcr3.2*, one of the three CXCR3 paralogs, in both *M. avium* and *M. marinum* infections [23]. Therefore, we sought to determine the role of Cxcr3.2 signaling in *M. avium* infection, utilizing *cxcr3.2*^*hu6044*^ mutant zebrafish. The results of *M. avium* infection in *cxcr3.2* mutants recapitulated previous results of *M. marinum* infection [20]. In that bacterial burden, total bacterial cluster count and average bacterial cluster size were all reduced in *cxcr3.2* mutants compared to the wild-type control (Fig. 5a–d). Additionally, in *cxcr3.2* mutant larvae, fewer macrophages were observed in the caudal hematopoietic tissue (CHT), the region where the granuloma-like aggregates are localized (Fig. 5e, f). Together, these findings suggest that although macrophages play a protective role in containing *M. avium* infection, their recruitment via Cxcr3.2 signaling may also facilitate bacterial dissemination, highlighting a dual role for macrophages similar to that observed in *M. marinum* infection despite the lack of ESX- 1 in *M. avium*.

### *M. avium* exhibits higher intracellular persistence than *M. marinum*

**Fig. 6:**
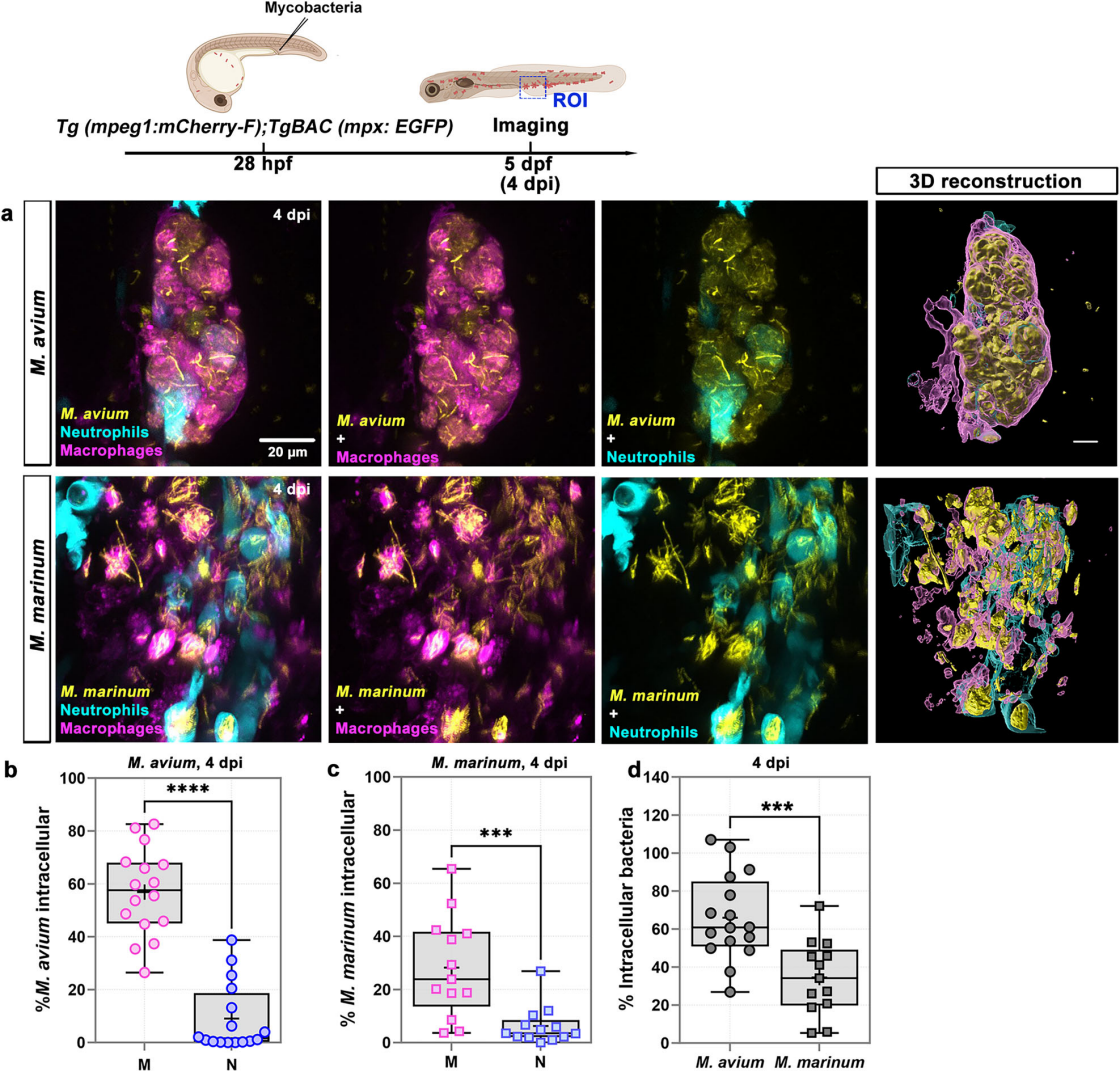
Residence of *M. avium* and *M. marinum* in macrophages and neutrophils during granuloma formation. *Tg(mpeg1: mCherry- F); TgBAC(mpx: EGFP)* larvae were systemically infected at 28 hpf with E2Crimson-labeled *M. avium* or *M. marinum*. **a** Representative images of the *M. avium* and *M. marinum*-infected *Tg(mpeg1: mCherry- F); TgBAC(mpx: EGFP)* larvae at 4 dpi. Scale bar: 20 µm. Scale bar is 10 µm in the 3D reconstruction panels. See Movie S5 and Movie S6 for more details of 3D reconstructions. **b**, **c** Comparison of *M. avium* (**b**) and *M. marinum* (**c**) distribution between macrophages and neutrophils at 4 dpi based on colocalization with *mpeg1:mCherry- F* and *mpx:EGFP* reporters. In (**b**, **c**), “M” means Macrophages; “N” means Neutrophils. **d** Percentage of intracellular *M. avium* and *M. marinum* at 4 dpi. In (**b**–**d**), data were combined from two independent experiments with 16 fish for *M. avium* infection and 13 fish for *M. marinum* infection (*n* = 16 for *M. avium* infection, *n* = 13 for *M. marinum* infection, *N* = 2). Statistical significance of difference was determined by two-tailed Mann–Whitney *U*-tests in (**b**, **c**) or unpaired *t*-test in (**d**). *** *P* < 0.001, **** *P* < 0.0001.

Having demonstrated different ways in which macrophages and neutrophils contribute to the containment and dissemination of *M. avium* infection, we sought to further investigate their role in the development of granulomas, the inflammatory structures typical of mycobacterial infections. Notably, *M. avium*-induced granuloma-like clusters appeared more compact than those formed during *M. marinum* infection (Fig. 6a), consistent with our previous findings [23]. The difference in structure was not due to a difference in the ratio of macrophage and neutrophil volume between *M. avium* infection and *M. marinum* infection (Fig. S6).

To determine if this difference in structure was due to better containment of *M. avium* infection, we quantified the percentage of *M. avium* and *M. marinum* residing within host cells (Fig. 6b–d; Movie S5 and Movie S6). At 4 dpi, both pathogens exhibited a preference for residing in macrophages over neutrophils (Fig. 6b, c). However, a significantly higher percentage of *M. avium* was found intracellular compared to *M. marinum* (Fig. 6d). These results suggest that *M. marinum* exhibits a greater tendency to dissociate from host cells by 4 dpi, whereas *M. avium* remains predominantly intracellular.

### *M. avium* induces less host cell death than *M. marinum*

**Fig. 7:**
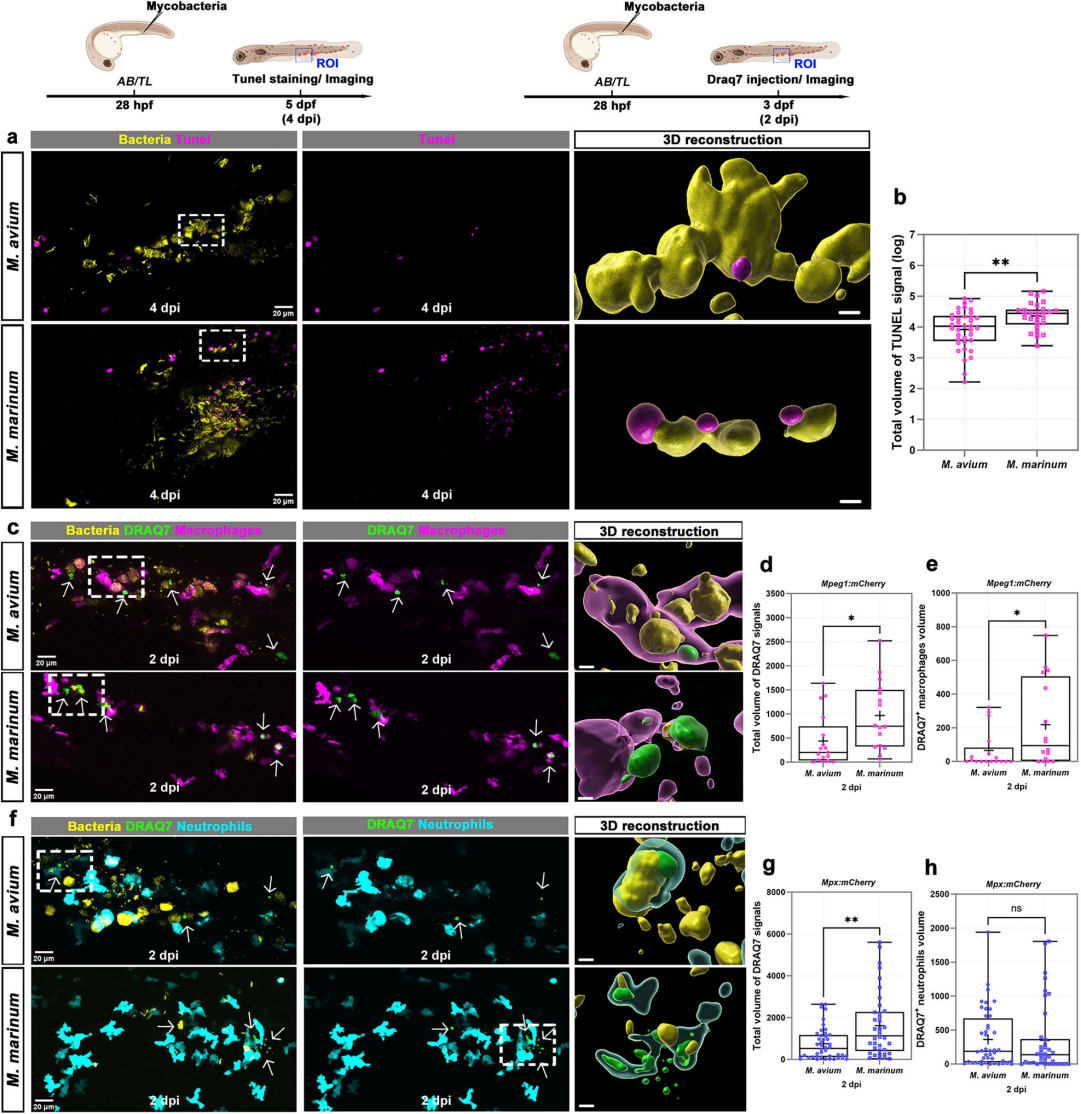
Activation of cell death pathways during *M. avium* and *M. marinum* infections. **a** Representative confocal images of TUNEL staining in *M. avium*-infected and *M. marinum*-infected larvae at 4 dpi. Scale bar: 20 µm. 3D reconstruction figures are on the right panel. Scale bar in 3D reconstruction images: 10 µm. **b** Quantification of total volume of TUNEL signals in mycobacteria- infected larvae at 4 dpi. Data were combined with four independent experiments (*n* = 37, 28, *N* = 4). In panel (**b**), data were log-transformed, and statistical significance of differences was determined by using Welch’s *t*-test, ** *P* < 0.01. **c** Representative confocal images showing DRAQ7-labeled macrophages in mycobacteria-infected larvae at 2 dpi. Scale bar: 20 µm. 3D reconstruction figures of the zoom-in area, squared by dashed boxes, are shown in the panels on the right. Scale bar in 3D reconstruction images: 10 µm. **d** Quantification of total volume of DRAQ7 signals in macrophage labeled-larvae at 2 dpi. **e** Quantification of DRAQ7 positive macrophage volume. **f** Representative confocal images showing DRAQ7-labeled neutrophils in mycobacteria-infected larvae at 2 dpi. White arrows point to the DRAQ7 signals in panels (**c**) and (**f**). In panels (**d**) and (**e**), data were combined from two independent experiments (*n* = 17, 16, *N* = 2). In panels (g and h), data were combined from three independent experiments (*n* = 44, 38, *N* = 3). Statistical significance of differences in panels (**d**, **e**, **g**, **h**) was determined by using two-tailed Mann–Whitney *U*-tests. * *P* < 0.05, **P < 0.01.

Having observed that *M. avium* exhibits a higher intracellular residence than *M. marinum*, we hypothesized that *M. avium* infection induces less cell death compared to *M. marinum* infection. Quantification of TUNEL signal volume as a measure for DNA fragmentation showed significantly higher levels of cell death in *M. marinum* infection compared to *M. avium* infection (Fig. 7a, b). To further characterize infection-associated cell death, we applied a cell-permeable dye DRAQ7 in transgenic zebrafish larvae with fluorescently labeled macrophages (Fig. 7c–e) or neutrophils (Fig. 7f–h). Consistent with the TUNEL assay, total DRAQ7 signals were markedly higher in *M. marinum*-infected larvae than in those infected with *M. avium* (Fig. 7c, d, f, g). Co-localization analysis revealed that DRAQ7⁺ macrophages were significantly more abundant in *M. marinum* infection (Fig. 7e), whereas no significant difference was observed in DRAQ7⁺ neutrophils between the two infections (Fig. 7h). Together, the TUNEL assay and DRAQ7 staining support the hypothesis that *M. avium* infection induces less cell death than *M. marinum* infection.

### Pyroptotic signaling plays opposing roles in *M. avium* and *M. marinum* infections

**Fig. 8:**
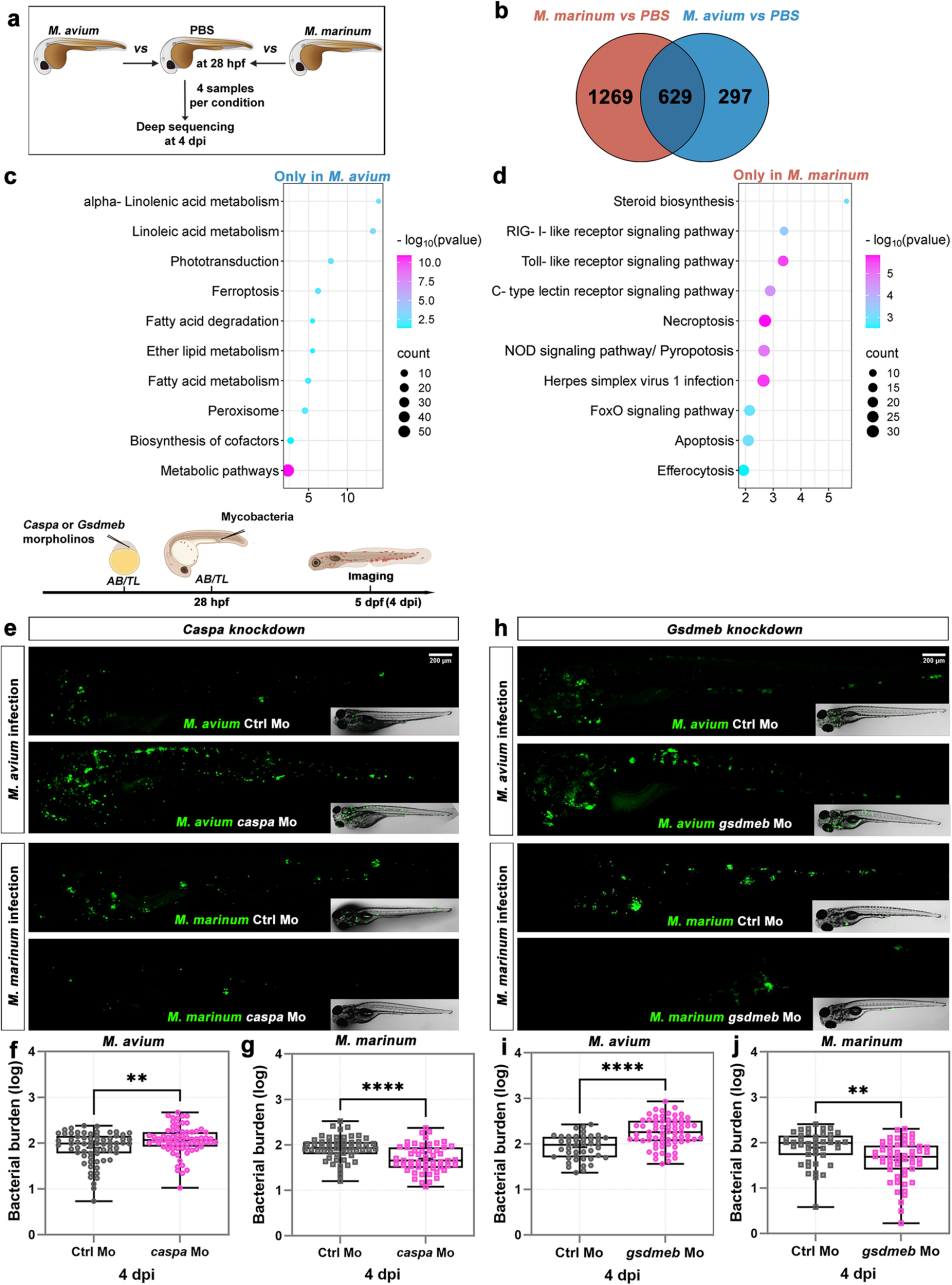
Opposing effects of knockdown of pyroptosis pathway genes during *M. avium* and *M. marinum* infection. **a** Experimental scheme of RNAseq samples collection as previously reported [23]. *AB/TL* embryos were systematically infected with *M. avium*, *M. marinum* and PBS (control) at 28 hpf, and larvae were collected for deep sequencing at 4 dpi. **b** The Venn diagram illustrates the number of shared and specific differentially expressed genes (DEGs) between *M. avium*-infected zebrafish larvae and *M. marinum*-infected zebrafish larvae in comparison to the PBS control. Genes with an FDR *P*-value < 0.05 were considered statistically significant. **c** Top 10 significantly enriched KEGG pathways from the DEGs specifically expressed in the *M. avium* infection group compared to the PBS control. **d** Top 10 significantly enriched KEGG pathways from DEGs specifically expressed in the *M. marinum* infection group compared to the PBS control. See Fig. S7 for the Top 10 significantly enriched KEGG pathways from the shared DEGs. In (**c**, **d**), the KEGG enrichment analyses were performed by using DAVID. The size of the circle represents the number of enriched genes, with larger circles indicating a higher number of enriched genes in the pathway. The color of the circle corresponds to −log10 (*P*-value), and the fold change is indicated on the *x*-axis. **e** Representative images for bacterial pixel count at 4 dpi following knockdown of *caspa* in *M. avium* (upper panel) and *M. marinum* (bottom panel)-infected larvae. Scale bar: 200 µm. **f**, **g** Quantification of bacterial burden at 4 dpi following knockdown of *caspa* in *M. avium* (**f**) and *M. marinum* (**g**) infected larvae. Data were combined from three independent experiments (*N* = 3). Sample size in (**f**) is 67, 72; sample size in (**g**) is 52, 52. **h** Representative images for bacterial pixel count at 4 dpi following knockdown of *gsdmeb* in *M. avium* (upper panel) and *M. marinum* (bottom panel)-infected larvae. Scale bar: 200 µm. **i**, **j** Quant**i**fication of bacterial burden at 4 dpi following knockdown of *gsdmeb* in *M. avium* (**i**) and *M. marinum* (**j**) infected larvae. Data were combined with three independent experiments (*N* = 3). Sample size in (**i**) is 44, 69; sample size in (**j**) is 43, 54. In panels (**f**, **g**, **i**, **j**), data were log-transformed, and statistical significance of differences was determined by using an unpaired *t*-test (**f**, **g**, **j**) or Welch’s *t*-test (**i**). **P < 0.01, ***P < 0.001, ****P < 0.0001.

In order to explore potential differences in cell death pathways, we re-analyzed our previously published transcriptome data [23]. We focused on the differentially expressed genes (DEGs) that were shared between the infections or specific for either *M. avium* or *M. marinum* infection (Fig. 8a, b and Fig. S7a, b). Functional pathway analysis using DAVID identified the top 10 significantly enriched KEGG pathways for the shared and pathogen-specific DEGs (Fig. S7c). Notably, apoptosis was enriched among the shared DEGs (Fig. S7c). However, distinct cell death pathways were observed between the two infections: *M. avium*-specific DEGs were only enriched in ferroptosis, whereas *M. marinum*-specific DEGs were associated with necroptosis, apoptosis, and NOD-like receptor signaling, a key upstream regulator of pyroptosis (Fig. 8c, d).

The TUNEL assay detects DNA fragmentation associated with apoptosis and pyroptosis, and NOD-like signaling/pyroptosis was one of the pathways differentiating *M. marinum* from *M. avium* infection in our transcriptome study [32]. Pyroptosis is a mode of programmed cell death that triggers inflammatory cytokine release upon cell lysis [13, 15]. Mycobacteria, including *M. tuberculosis* and *M. marinum*, exploit pyroptosis to facilitate bacterial dissemination within the host [13, 16]. The inflammasome-mediated induction of caspase-1 (CASP1) activity is required for maturation of pro-IL-1β and cleavage of the pore-forming protein gasdermin D (GSDMD), which executes pyroptosis [33, 34]. In zebrafish, Caspase a (Caspa) functions as a homolog of mammalian CASP1, promoting the maturation of Gasdermin Eb (Gsdmeb) [16, 35]. Consistent with previous findings [16], knockdown of *caspa* or *gsdmeb* significantly reduced bacterial burden in *M. marinum*-infected larvae (Fig. 8e, g, h, j). Interestingly, the opposite effect was observed in *M. avium* infection, where bacterial burden increased following *caspa* or *gsdmeb* knockdown (Fig. 8e, f, h, i). These results suggest that while pyroptotic signaling is detrimental during *M. marinum* infection, it plays a host-protective role during *M. avium* infection.

## Discussion

Infections with non-tuberculous mycobacterial pathogens, notably *M. avium*, are notorious in immunocompromised patients, where treatment options are complicated by antibiotic resistance as well as limited understanding of the pathogenic mechanisms. A fundamental difference between *M. avium* and other well-studied mycobacteria is the absence of ESX-1, a secretion system that is essential for the virulence and intercellular survival of species like *M. tuberculosis* and *M. marinum*. In this study, we employed previously established *M. avium* and *M. marinum* infection models in the zebrafish host to investigate how these pathogens differ in establishing pathogenesis. Despite the absence of ESX-1, *M. avium* exhibited several parallels in virulence with *M. marinum*, including the ability to exploit macrophages and neutrophils as vehicles for expansion and dissemination. Furthermore, the host defense response to both pathogens was similar regarding the crucial role of macrophages in restricting mycobacterial growth. The key difference revealed by our study is the propensity of *M. avium* and *M. marinum* infections to induce cell death pathways. While lytic cell death of macrophages, including pyroptosis, exacerbates *M. marinum* infection, we found that cell death was less frequent during *M. avium* infection, and we could demonstrate a protective role for pyroptosis in the response to this pathogen. Collectively, these findings reveal both conserved and distinct host immune responses to *M. avium* and thereby offer new insights into the mechanisms underlying *M. avium* pathogenesis.

### Shared features of the immune response in *M. avium* and *M. marinum* infections

Despite its lower virulence compared to *M. marinum* [23], *M. avium* is still capable of triggering hallmark immune responses, including granuloma formation and strong pro-inflammatory activation. Among proinflammatory cytokines, TNFα is essential for protective immunity in human TB patients, although excess TNFα levels may contribute to pathological inflammation [36]. In agreement, resistance to mycobacterial infections is impaired in *tnfa*/TNFα-deficient zebrafish and mice, or when Tnfa/TNFα is overproduced [25, 37–39]. By combining gene expression analysis with live imaging of the activity of a fluorescent *tnfa* reporter, we demonstrate that the majority of *M. avium-*infected macrophages mount a robust *tnfa* response, which correlated with the infection volume of individual macrophages. Thus, *tnfa* expression associated with proinflammatory polarization of macrophages is a shared feature of *M. marinum* and *M. avium* infections in zebrafish, which parallels the human immune responses to *M. tuberculosis* and *M. avium* infections [36, 40].

Macrophages are crucial responders to *M. tuberculosis* and *M. marinum* infections [29], but the role of neutrophils in mycobacterial defense varies by tissue [9, 12, 24]. While they are generally protective—evidenced by increased bacterial burden when their recruitment is inhibited—their direct interaction with *M. marinum* is not observed in early systemic infections [12]. However, in localized infections, neutrophils actively phagocytose *M. marinum* and contribute to its dissemination [9]. In this study, we found that macrophage-depleted zebrafish embryos were more susceptible to *M. avium* infection, confirming that macrophages are essential for the host to survive infection with this pathogen. Macrophage depletion has also been shown to increase bacterial burden or cause lethality in *M. marinum*, *M. kansasii*, *M. abscessus*, and *M. fortuitum* infections [29, 41–44]. Together, these studies support that the protective function of macrophages is shared between different NTM infections. However, the role of alveolar macrophage depletion in *M. tuberculosis* infection remains controversial [45–47]. While some mouse studies reported lower bacterial counts at later stages of infection (five weeks post-infection) following macrophage depletion [45]. Others found that genetic ablation of alveolar macrophages using GM-CSF-deficient mice resulted in increased *M. tuberculosis* burden [47]. In contrast to *M. marinum* and *M. abscessus* infections in the zebrafish model [9, 12, 37]. where neutrophils play an essential role, we did not observe major changes in either survival rates or bacterial burden upon *M. avium* infection in neutrophil-depleted zebrafish larvae. The reverse migration of *M. avium-*infected neutrophils that we observed by live imaging suggests that neutrophils contribute to infection dissemination rather than serving a protective role. We further provide evidence that macrophages restrict the potentially detrimental role of infected neutrophils by engulfing these cells, further supporting the importance of macrophages for host defense against *M. avium*.

Notwithstanding the crucial defense function of macrophages, mycobacteria can exploit macrophages for spreading between cells and tissues [10, 20, 29]. This behavior of macrophages is driven by the ESX-1 secretion apparatus of *M. tuberculosis* and *M. marinum*, but this virulence system is not present in *M. avium* [5]. Nevertheless, our results suggest that macrophages can still facilitate the spreading of *M. avium* infection. Previously, we demonstrated that *cxcr3.2*, encoding a homolog of the human CXCR3 chemokine receptor in zebrafish, is upregulated in both *M. avium* and *M. marinum* infections [23]. Additionally, our previous studies showed that *cxcr3.2* plays a role in macrophage recruitment and motility during *M. marinum* infection [20, 31, 48]. Consistently, in this study, we found that *cxcr3.2* is also required for macrophage recruitment in *M. avium* infection. Moreover, in both *M. avium* and *M. marinum* infections, the absence of *cxcr3.2* led to decreased granuloma formation and attenuated infections, indicating that Cxcr3.2-mediated macrophage migration promotes the pathogenesis of both mycobacterial species. In addition, disrupting *cxcr3.2* in zebrafish during *M. marinum* infection results in increased lysosomal staining and upregulation of lysosome-related genes, suggesting that enhanced bactericidal activity may also contribute to the lower *M. avium* burden observed here [31]. Together, our results show that, while macrophages are essential for early containment of *M. avium* or *M. marinum*, their recruitment via *cxcr3.2* also promotes bacterial expansion and dissemination, emphasizing a dual role for macrophages in infection dynamics that exists independently of ESX-1 virulence.

### Opposite roles of inflammatory cell death in *M. avium* and *M. marinum* infections

A prominent difference between *M. avium* and *M. marinum* infections, which emerged from our study, relates to the occurrence of cell death in granulomas. While abundant cell death was detected in *M. marinum* granulomas, macrophages in *M. avium* granulomas survived with high bacterial loads, and cell death was relatively rare. These differences were reflected in the transcriptomics signatures, where *M. marinum* but not *M. avium* infection elicited the expression of genes involved in lytic cell death pathways, including necroptosis and pyroptosis. Although these pathways serve as defense mechanisms against various bacterial and viral infections, mycobacteria have evolved subversion strategies [13, 49–51]. In the zebrafish *M. marinum* model, necroptosis has been shown to exacerbate pathogenesis under inflammatory conditions where Tnfa was experimentally elevated, while pyroptosis was found to exacerbate disease in both wild-type and autophagy-deficient larvae [16, 39]. Likewise, there is growing evidence that *M. tuberculosis* exploits pyroptosis to promote inflammation and bacterial dissemination [52, 53].

Notably, our study revealed that genetic inhibition of pyroptosis has an opposite effect on *M. marinum* and *M. avium* infections. Based on knockdown of *caspa* and *gsdmeb*, we found that activation of the pyroptosis pathway is detrimental for resistance to *M. marinum*, yet serves a protective function towards *M. avium*. This protective effect of pyroptosis could be mediated by its inflammatory signaling function, such as IL-1β secretion, rather than by cell lysis itself. Additionally, pyroptotic lysis could disrupt the bacterial replicative niche and expose *M. avium* to soluble antimicrobial molecules, as demonstrated for other pathogens [54]. Furthermore, pyroptosis triggers pore-induced intracellular traps (PITs), which immobilize bacteria and facilitate their clearance through efferocytosis [55]. Finally, GSDMD has been shown to directly eliminate bacteria by forming pores in their membranes [34, 56]. The limited occurrence of cell death during *M. avium* infection could explain why the protective effects of pyroptosis predominate, whereas abundant cell death in *M. marinum* granulomas would drive excess inflammation, exacerbating bacterial growth.

In conclusion, in this study, we addressed the poorly understood mechanisms of NTM disease by investigating the pathogenesis of *M. avium* infection using zebrafish larvae as an in vivo infection model in comparison with the widely used *M. marinum* tuberculosis model. Our findings highlight both shared and distinct host-pathogen interaction mechanisms between these two mycobacterial species. We demonstrate that, during the early stages of infection, macrophages play a crucial role in controlling *M. avium* infection, while neutrophils primarily contribute to bacterial dissemination. Additionally, we found that *M. avium* induces a strong proinflammatory response in macrophages, marked by high *tnfa* expression, even in the absence of ESX-1, a major virulence factor in *M. marinum* and *M. tuberculosis*. The activation of macrophage migration by *cxcr3.2*-mediated chemokine signaling represents an ESX-1- independent mechanism exploited by both *M. avium* and *M. marinum* for expansion in granulomas. Notably, unlike in *M. marinum* and *M. tuberculosis* infections, where pyroptosis exacerbates disease progression, our results show that genes in the pyroptosis pathway, including *caspa* and *gsdmeb*, play a protective role in *M. avium* infection. These findings provide new insights into the immune mechanisms underlying *M. avium* infection and highlight key differences with other mycobacterial pathogens.

## Material and methods

### Zebrafish

Zebrafish husbandry and experimentation were approved by the local animal Ethics Committee (“Dierexperimentencommissie - DEC”) of Leiden University (License number: AVD10600202216175) and adhered to the guidelines of the Leiden University Animal Welfare Body. All procedures complied with the international standards outlined in the EU Animal Protection Directive 2010/63/EU. Adult zebrafish were not sacrificed in this study and were used solely for breeding purposes. Experiments were limited to zebrafish larvae up to 5 dpf, before the free-feeding stage. Zebrafish larvae experiments were conducted according to standard protocols available at http://www.zfin.org/. Eggs and larvae were raised at 28.5 °C in egg water containing 60 g/mL Instant Ocean sea salts. For all experiments, larvae younger than 5 dpf were anesthetized with egg water supplemented with 0.02% buffered 3-aminobenzoic acid ethyl ester (Cat#886-86-2, Tricaine, Sigma-Aldrich, Netherlands).

The zebrafish strains used in this study included: AB/TL wild-type zebrafish strain, *cxcr3.2*^*hu6044*^ homozygous mutant (*cxcr3.2*^*-/-*^) and the offspring of their wild-type siblings (*cxcr3.2*^*+/+*^) [20]. Additionally, we used double fluorescent lines *Tg (mpeg1:mCherry-F);TgBAC (mpx:EGFP)* [37]. *Tg(Mpeg:Gal4:UAS:NTR:mCherry)* (referred to as *mpeg1/* NTR^*+*^ in this study) for macrophage ablation, *Tg(mpx:Gal4-VP16/ UAS- E1b:nfsBmCherry*^*i149*^*)* (referred to as *mpx/* NTR^+^ in this study) for neutrophil ablation [27]. and *Tg (mfap4:mCherry-F/ TNFa:GFP-F)* for M1 macrophage visualization [26].

### Morpholino injection

Previously validated morpholino oligonucleotides (MOs) targeting *caspa* and *gsdmeb* were custom-synthesized by Gene Tools (Gene Tools, USA) [16]. The sequence of the *caspa* MO is 5′-GCCATGTTTAGCTCAGGGCGCTGAC-3′, and the *gsdmeb* MO sequence is 5′-TCATGCTCATGCTAGTCACCCACC-3′. MOs were dissolved in Milli-Q water supplemented with 0.05% phenol red to aid visualization during injection. A volume of 1 nL of either *caspa* MO (0.6 mM) or *gsdmeb* MO (0.7 mM) was microinjected into the yolk of zebrafish embryos at the 1–2 cell stage, according to previously established methods [16].

### Bacterial cultures and infections in zebrafish

The *M. marinum* M-strain expressing mWasabi fluorescent protein [57]. The *M. avium Chester* (also called MAC 101, ATCC® 700898™) expressing mWasabi fluorescent protein via the expression vector pSMT3, or E2crimson via pTEC19, were cultured and prepared for zebrafish infection as described previously [23]. Briefly, embryos were systemically injected with 4500 CFU fluorescent protein-labeled *M. avium* or 250 CFU of fluorescent protein-labeled *M. marinum* via caudal vein at 28 hpf, leading to comparable levels of bacterial burden at 4 dpi. For local infection, ~50 CFU of *M. avium* was injected into the tail fin at 3 dpf.

### Inducible genetic ablation of macrophages or neutrophils

To investigate the role of macrophages or neutrophils in *M. avium*-infected zebrafish larvae, metronidazole (Cat# 288-32-4, MTZ, Sigma-Aldrich) treatment was used for cell ablation as previously described [26, 27]. The *Tg(Mpeg:Gal4:UAS:NTR:mCherry)* line was used for macrophage ablation, while *Tg(mpx:Gal4-VP16/ UAS-E1b:nfsBmCherry*^*i149*^*)* line was used for neutrophil ablation. Manually dechorionated zebrafish larvae were incubated in E3 medium containing 5 mM MTZ for 1 h before microinjection with *M. avium* or PBS control. Infected zebrafish larvae were maintained in E3 medium containing 5 mM MTZ. The depletion efficiency of macrophages or neutrophils was assessed at 4 dpf. The survival rate of macrophage- or neutrophil-ablated zebrafish larvae and their control groups was quantified at 1, 2, 3, and 4 dpi. The bacterial burden of macrophage- or neutrophil- ablated zebrafish larvae was quantified at 3 dpi.

### Chemical ablation of macrophages

For ablation of circulating monocytes/macrophages, 5 nL of liposome-encapsulated clodronate (Lipo-C; LIPOSOMA, Netherlands) was injected into zebrafish larvae via the duct of Cuvier at either 1 dpi or 3 dpi with *M. avium* [28]. Control larvae were injected with 5 nL of liposome-encapsulated PBS (Lipo-PBS; LIPOSOMA, Netherlands). The efficiency of macrophage depletion was assessed at 4 dpf for early ablation and at 5 dpf for late ablation. *M. avium* burden was quantified at 3 dpi for early macrophage ablation and at 4 dpi for late ablation.

### TUNEL assay

Terminal deoxynucleotidyl transferase dUTP nick- end labeling (TUNEL) staining was used to detect cell death in 4 dpi larvae following *M. avium* or *M. marinum* infections. The In Situ Cell Death Detection Kit, TMR Red (Cat# 12156792910, Roche, Switzerland) was used according to the manufacturer’s instructions. The assay was performed as previously described [16]. Samples were stored in PBST (PBS containing 0.1% Tween 20) for confocal microscopy as mentioned below.

### DRAQ7 dye injection

DRAQ7 dye (Cat# D15105, Invitrogen, Netherlands) was used to detect cell death in zebrafish larvae at 2 dpi with *M. avium* or *M. marinum*. DRAQ7 is a far-red fluorescent dye that selectively binds to nuclear DNA in cells that have lost plasma membrane integrity. A total of 2 nL of 0.3 mM DRAQ7 solution was injected into the duct of Cuvier of infected larvae at 2 dpi. Confocal imaging was conducted 2 h after DRAQ7 administration to visualize and quantify DRAQ7-positive cells.

### Imaging and quantification of bacterial burden

Mycobacteria-infected fish in Figs. 1, 4, 5a, and 8 were imaged by using a fluorescence stereomicroscope (Leica M205FA) equipped with a DFC 345FX color camera (Leica Microsystems). All experiments were independently performed at least twice, and images from independent experiments were captured using the same microscope settings. Bacterial pixel counts, cells pixel counts, bacterial cluster size, and cluster numbers in Figs. 1, 4, 5, and 8 were analyzed by using Quantifish software (https://github.com/DavidStirling/QuantiFish) [58].

### Confocal microscopy imaging

Confocal microscopy was utilized to observe leukocyte migration behaviors (Fig. 2), *tnfa* expression (Fig. 3), granuloma-like cluster formation in the absence of *cxcr3.2* (Fig. 5e), macrophage and neutrophil responses (Fig. 6), the TUNEL assay (Fig. 7a), and DRAQ7 signal detection (Fig. 7c, f) following mycobacterial infection. Observed larvae were anesthetized with 0.02% tricaine and subsequently immobilized in 1% low melting-point agarose (Cat# 9012-36-6, SERVA, Germany). Images were acquired using a Leica TCS SP8 confocal microscope (Leica Microsystems). Blood island-infected larvae or tail fin-infected larvae were imaged with a 20× objective (N.A. 0.75) (Fig. 3a, b, e, Fig. 5, and Fig. 6a). To study cell death, images of fixed 4 dpi larvae in Fig. 7a were acquired with a 40× objective (oil immersion, N.A. 1.3). To detect DRAQ7 signal in mycobacteria-infected larvae at 2 dpi, images were acquired with a 20× objective (N.A. 0.75). The total volume of TUNEL staining or DRAQ7 signal in the CHT region was quantified using Fiji/ImageJ (Fig. 7). Representative images in this study are shown as maximum projections. The Surface tool of Imaris software (version 10.2) was used to reconstruct the 3D images (Fig. 2a, Fig. 3b, f, Fig. 6a and Fig. 7a, c, f).

### Living imaging, cell tracking, and quantification

Time-lapse imaging in Fig. 2 was performed on 3 dpf zebrafish larvae. The larvae were mounted into 1.5% low melting- point agarose (Cat# 9012-36-6, SERVA, Germany) after being anesthetized with 0.02% triciane. Time-lapse images were acquired using a Leica TCS SP8 confocal microscope (Leica Microsystem) with 1 min time interval for 9.5 h with 20× objective (N.A. 0.75). Cell tracking of leukocytes upon *M. avium* infection was performed using the manual tracking plug-in from Fiji [20, 59]. The data on the leukocyte velocity were output from the manual tracking data table.

### RNAseq analysis

To study differences in enriched cell death pathways, the RNAseq data from our previous study (available at NCBI GEO, GSE218892) were used [23]. An FDR *P*-value < 0.05 was set as the cutoff to define significantly regulated genes. The Venn diagrams were generated via the website: https://www.biovenn.nl/. KEGG enrichment analysis was conducted by using DAVID (http://david.abcc.ncifcrf.gov/summary.jsp).

### Statistical analyses

Although no statistical method was used to predetermine sample size, the sample size used in this study was based on a previous publication in which power calculations were performed and found sufficient to detect an effect size of at least 20% [16, 26]. The larvae with improper injections were excluded prior to analysis; no samples were excluded afterward. Sample size and number of biological replicates are mentioned in the figure legends. Blinding was employed during experiments and data analysis when possible. Statistical analyses in this study were performed by using Graphpad Prism software (version 10.1.2; San Diego, CA, United States). The Shapiro–Wilk test was used to assess whether data followed a normal (Gaussian) distribution. Normally distributed data were analyzed using unpaired or paired (Fig. 1f) two-tailed t-tests for two-group comparisons. Detailed information is shown in each figure legend. For data that did not follow a normal distribution, two approaches were applied depending on the sample size characteristics. When group sizes were comparable, statistical significance was determined using unpaired Mann–Whitney *U*-tests for two-group comparisons, or Kruskal–Wallis one-way analysis followed by Dunn’s multiple comparison test for comparisons involving more than two groups. When sample sizes differed substantially between groups (Figs. 7b and 8i), data were log-transformed and analyzed using Welch’s *t*-test. Boxplots show individual data points, and their boxes indicate the interquartile range, with the median as a horizontal line and the mean as a plus symbol (+). Whiskers represent the minimum and maximum values. 25% Percentile, medians, 75% percentile and the interquartile range (IQR) are shown in Table S1.

## Supplementary information

Movie S1 Time-lapse imaging of M. avium-infected neutrophils are engulfed by macrophages.

Movie S2 Time-lapse imaging of neutrophil reverse migration following M. avium infection in the tail fin.

Movie S3 Macrophage tracking in Tg(mpeg1:mCherry- F);TgBAC(mpx:EGFP) larvae infected with M. avium in the tail fin.

Movie S4 Neutrophil tracking in Tg(mpeg1:mCherry- F);TgBAC(mpx:EGFP) larvae infected with M. avium in the tail fin.

Movie S5 3D reconstruction of Tg(mpeg1:mCherry- F);TgBAC(mpx:EGFP) larvae systemically infected with M. avium at 4 dpi.

Movie S6 3D reconstruction of Tg(mpeg1:mCherry- F);TgBAC(mpx:EGFP) larvae systemically infected with M. marinum at 4 dpi.

Supplementary Information

Supplementary Table 1

## Data Availability

The repository of the images in this study is available at BioImage Archive: 10.6019/S-BIAD2463. The other datasets used and analyzed during the current study are available from the corresponding author on reasonable request.
