# Supplementary material for: Protective versus deleterious roles of pyroptosis in Mycobacterium avium and Mycobacterium marinum infections
Source: Cell Death Dis. 2026 Apr 30;17(1):580. doi: 10.1038/s41419-026-08810-1 (PMC13275888; doi:10.1038/s41419-026-08810-1)
Supplement: Supplementary file 7 — Supplementary Information [file 41419_2026_8810_MOESM7_ESM.docx]

**Supplementary material**

Protective versus Deleterious Roles of Pyroptosis in *Mycobacterium avium* and *Mycobacterium marinum* Infections

Wanbin Hu^1^, Federico Pagliaro^1^, Herman P. Spaink^1^, Annemarie H. Meijer^1*^

^1^Institute of Biology Leiden, Leiden University, Einsteinweg 55, 2333 CC Leiden, The Netherlands

***Corresponding author:** Annemarie H. Meijer

Address: Institute of Biology Leiden, Leiden University, Einsteinweg 55, 2333 CC, Leiden, The Netherlands

Telephone: +31 71 527 4927

Email: [a.h.meijer@biology.leidenuniv.nl](mailto:a.h.meijer@biology.leidenuniv.nl)

**COMPETING INTERESTS**

The authors declare no competing interests.


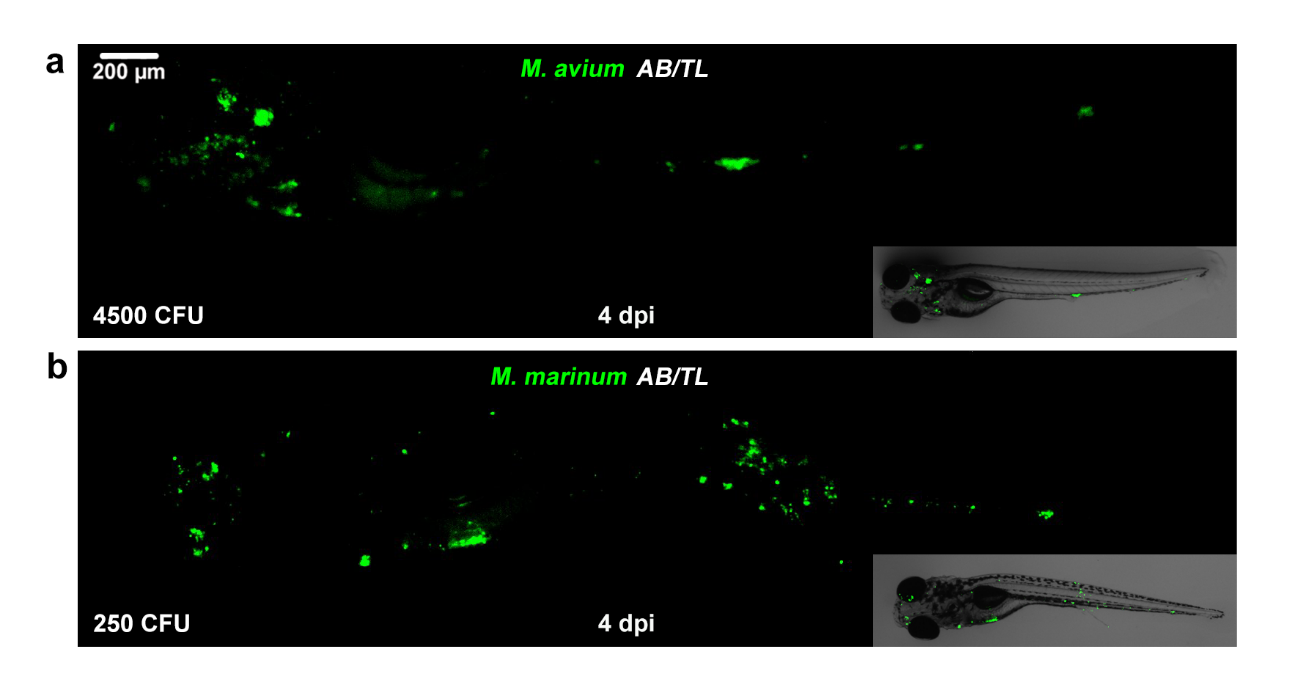


**Fig. S1 Representative images of *M. avium* infected- and *M. marinum* infected-zebrafish larvae.** **a** *M. avium*-infected zebrafish larvae at 4 dpi. ~4500 CFU *M. avium* MAC 101 strain was injected in zebrafish embryos at 28 hpf. **b** *M. marinum*- infected zebrafish larvae at 4 dpi. ~250 CFU *M. marinum* M strain was injected in zebrafish embryos at 28 hpf. Scale bar: 200 µm.


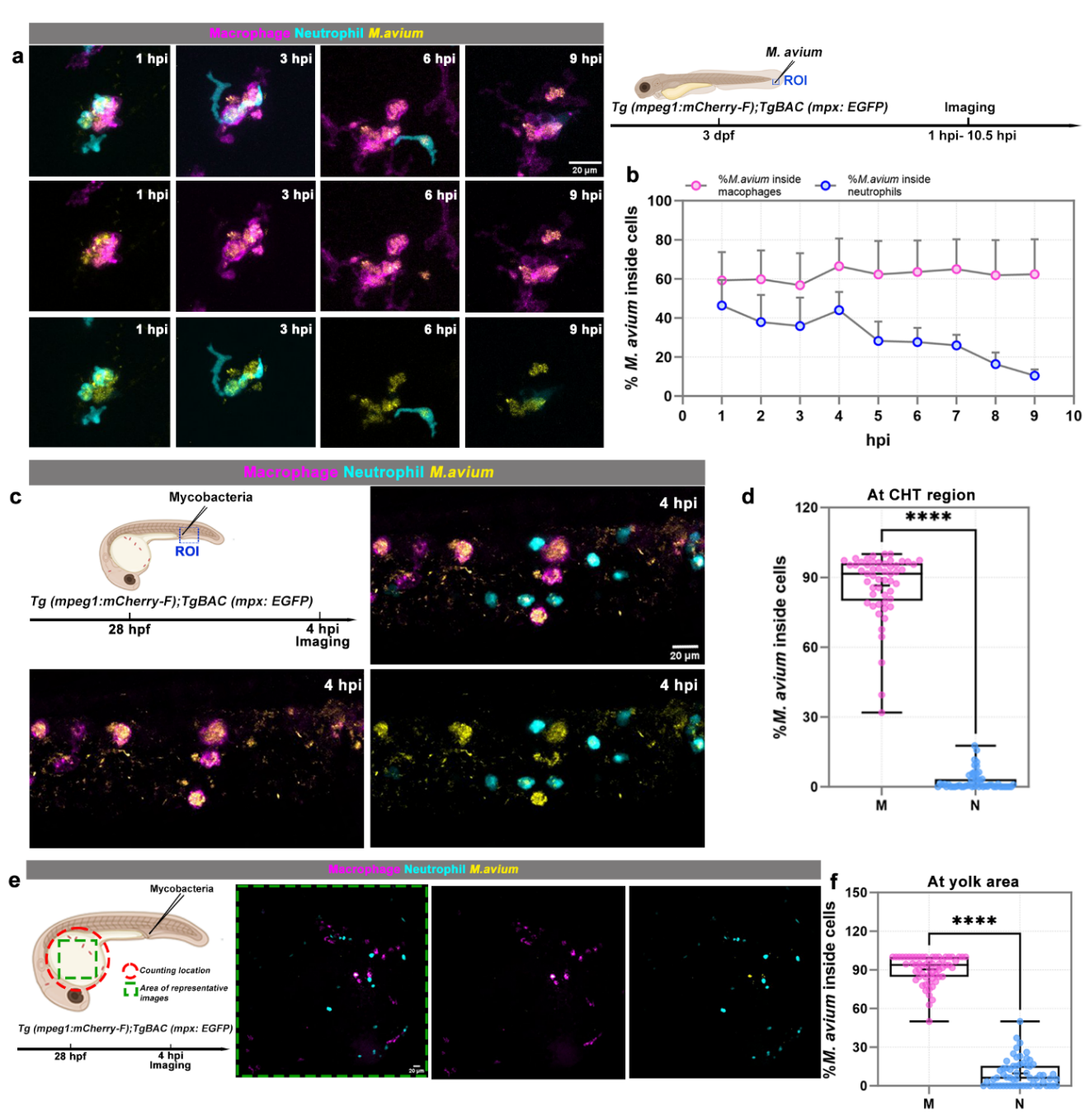


**Fig. S2 Residence of *M. avium* in macrophages and neutrophils at early infection stages**. *Tg(mpeg1:mCherry-F);TgBAC(mpx:EGFP)* embryos were infected with E2Crimson-labeled *M. avium* strain MAC 101 either locally or systemically. For local infections, embryos were infected at 3 dpf with ~50 CFU by tail fin injection. For systemic infections, embryos were injected with ~4,500 CFU *M. avium* via the blood island, and the caudal hematopoietic tissue (CHT) or yolk regions were analyzed. (a) Representative images showing interactions between different phagocytes and *M. avium* in the tail fin at 1, 3, 6, and 9 hpi. (b) Quantification of *M. avium* residing within macrophages or neutrophils. (c) Representative images showing phagocyte –*M. avium* interactions in the CHT region at 4 hpi. (d) Percentage of *M. avium* residing within macrophages or neutrophils in the CHT at 4 hpi. (e) Representative images of phagocyte –*M. avium* interactions in the yolk area at 4 hpi. Red dashed circles mark the quantified region; the green dashed box indicates the magnified area shown in (f). (f) Percentage of intracellular *M. avium* in macrophages or neutrophils at the yolk region at 4 hpi. In (b), data are presented as mean ± SEM (N = 5). In (d, f), data represent two independent experiments (n = 53, n = 61). Statistical significance was determined using unpaired Mann–Whitney U tests. *****P* < 0.0001. Scale bar: 20 µm.


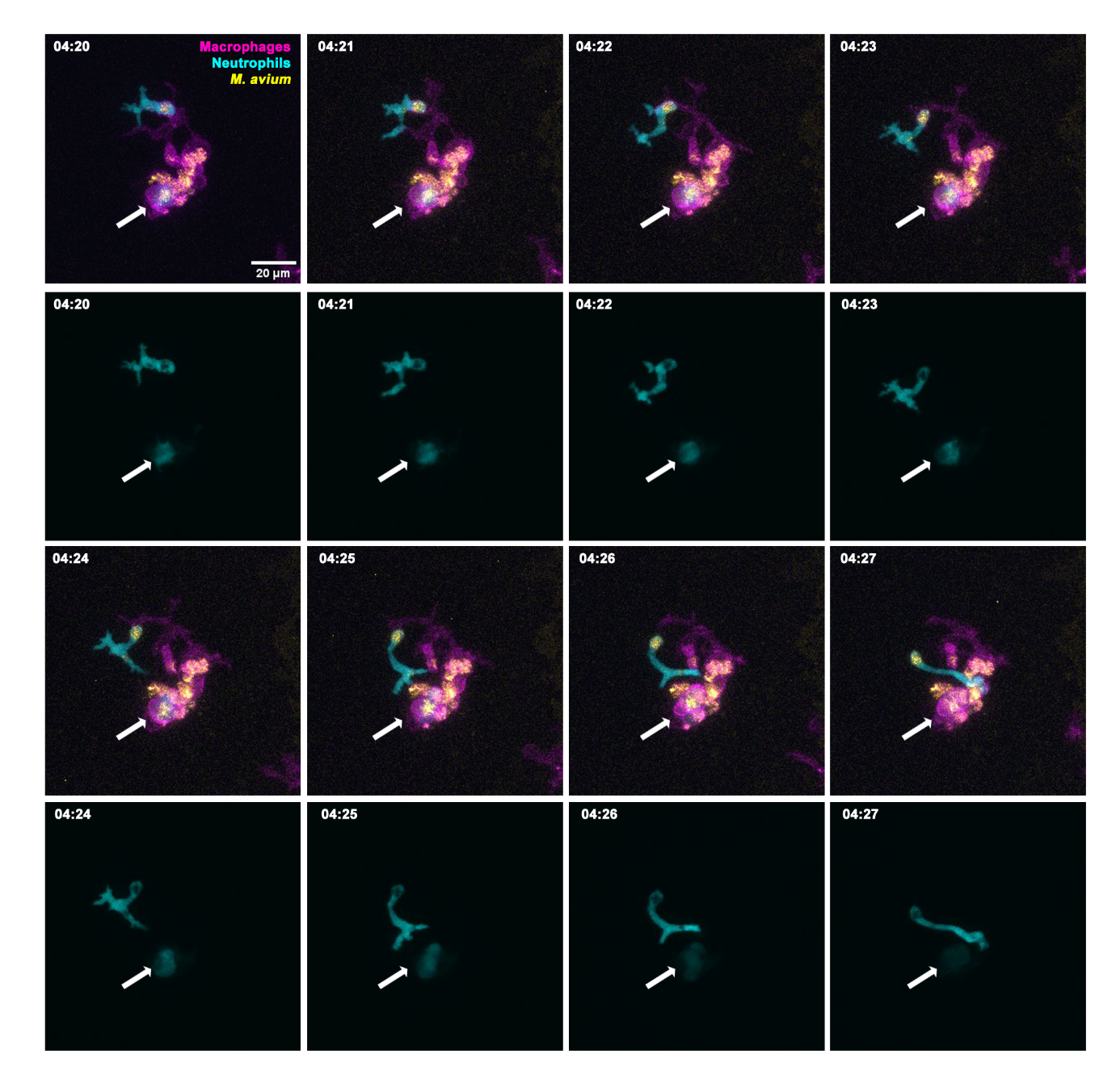
 **Fig. S3** **Time-lapse imaging of an infected neutrophil engulfed by a macrophage during *M. avium* infection.** *Tg(mpeg1:mCherry-F);TgBAC(mpx:EGFP)* zebrafish larvae were locally infected in the tail fin with E2Crimson-labeled *M. avium* strain MAC 101. Shown are representative images from eight consecutive time points illustrating a macrophage engulfing an *M. avium*-infected neutrophil. Magenta indicates macrophages, cyan indicates neutrophils, and yellow indicates *M. avium*. Scale bar: 20 µm.


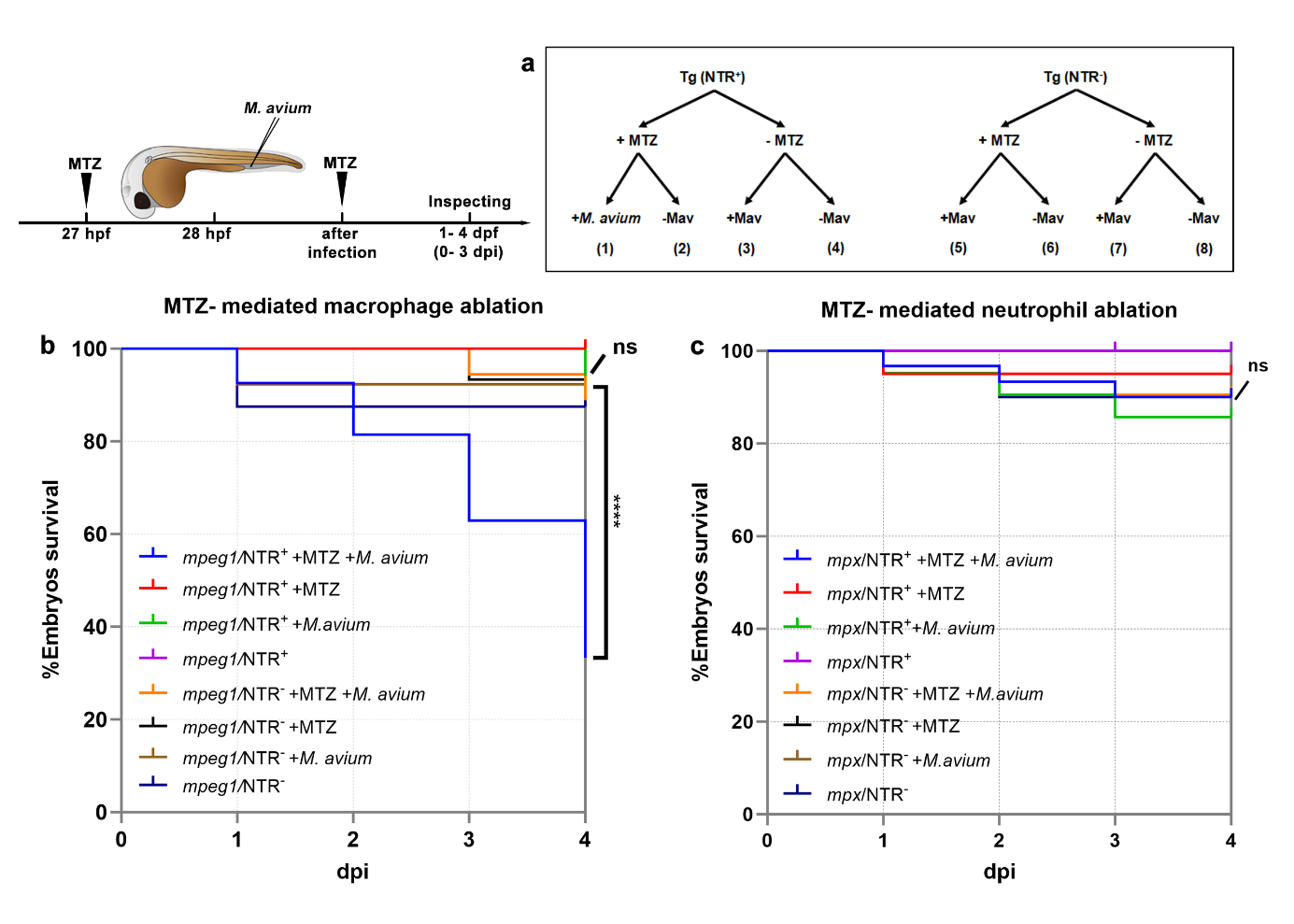
**Fig. S4 Percentage of surviving embryos with *M. avium* infection in macrophage-ablated, neutrophil-ablated and control groups. a** Groups in the cell ablation experiments. To investigate the role of macrophages and neutrophils in defence against *M. avium* infection, group (1) and group (3) were compared. To test the efficient of MTZ treatment, group (2) and group (4) were compared. To understand if MTZ treatment has an effect on *M. avium* growth, group (5) and group (7) were used. To determine whether MTZ treatment is non-toxic, group (6) and group (8) were used. **b** Percentage of survival embryos in macrophage ablation group and the other controls. **c** Percentage of survival embryos in neutrophil ablation group and the other controls. For simplicity, only groups 1, 3, 5 and 7 are displayed in the main figure (Fig. 4g, h).

**
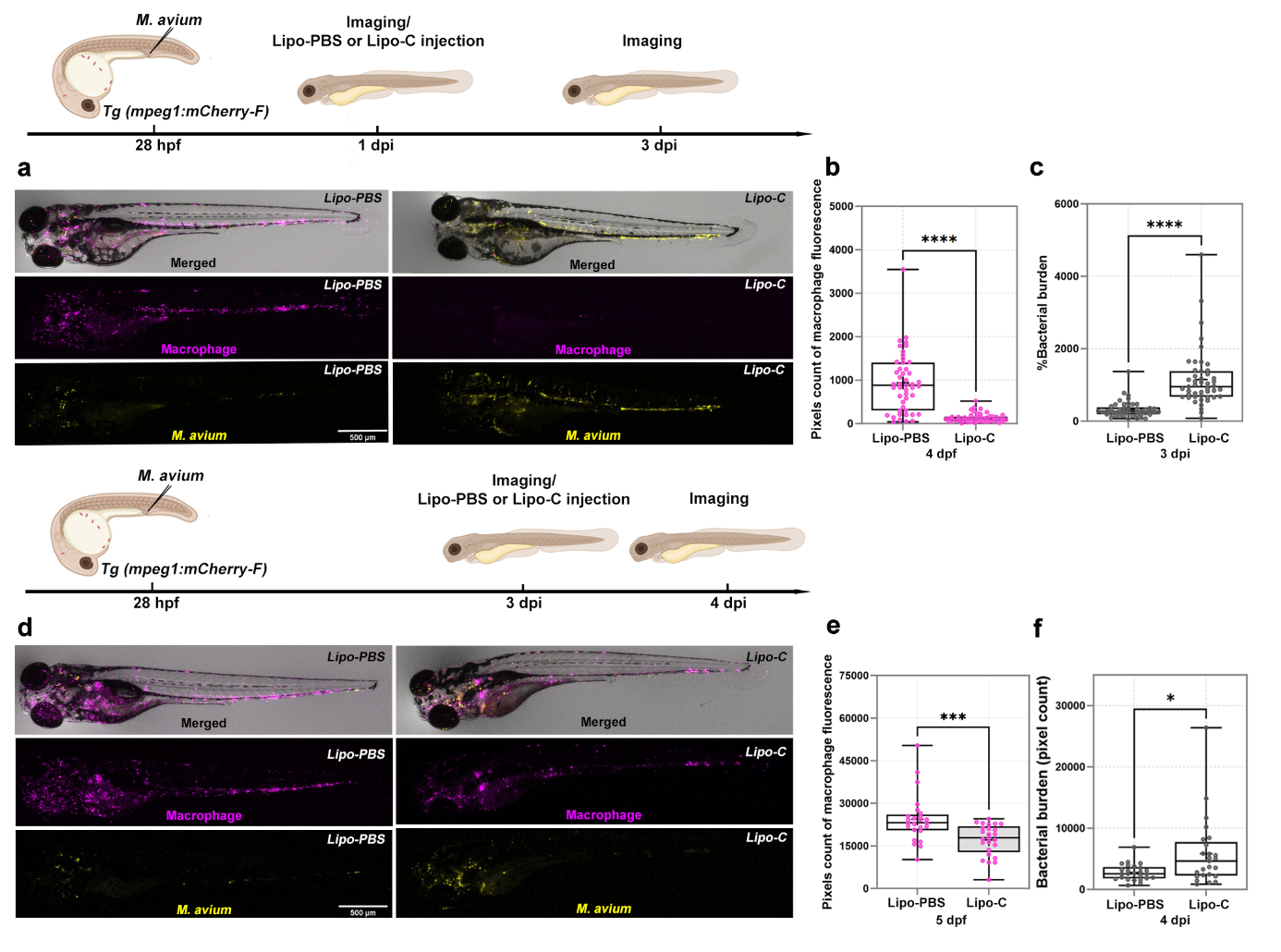
**

**Fig. S5** **Liposome clodronate–mediated macrophage depletion at early and late infection stages. a–c** Early macrophage ablation: *Tg(mpeg1:mCherry-F)* zebrafish larvae were injected with liposome clodronate (Lipo-C) or liposome PBS (Lipo-PBS) via the duct of Cuvier at 1 day post-infection (dpi). **a** Representative fluorescence images of Lipo-PBS- and Lipo-C-injected larvae at 3 dpi. **b** Quantification of macrophage fluorescence intensity (pixel counts) at 4 days post-fertilization (dpf). **c** Quantification of bacterial burden at 3 dpi. d-f Late macrophage ablation: larvae were injected with Lipo-C or Lipo-PBS at 3 dpi. In (b, c), data represent two independent experiments (n = 47, n = 47). **d** Representative fluorescence images of Lipo-PBS- and Lipo-C-injected larvae at 4 dpi. **e** Quantification of macrophage fluorescence intensity (pixel counts). **f** Quantification of bacterial burden at 4 dpi. Scale bar in (a, d): 500 µm. In (e, f), data represent two independent experiments (n = 25, n = 25). Statistical significance was determined using unpaired Mann–Whitney U tests. **P* < 0.05, ****P* < 0.001, *****P* < 0.0001. Scale bar: 20 µm.


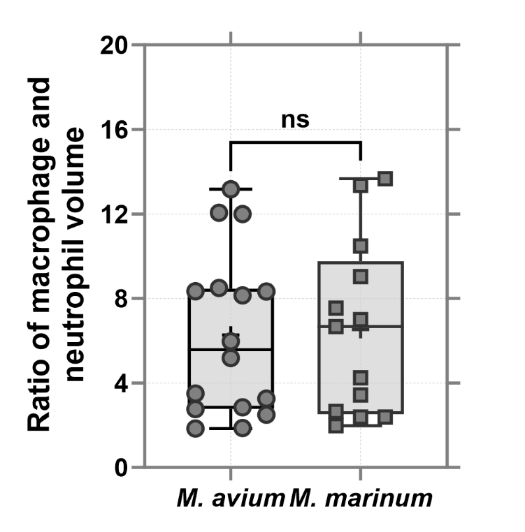


**Fig. S6 Ratio of macrophage and neutrophil volume at the infected area in zebrafish larvae.** Macrophage and neutrophil volumes were analyzed based on *mpeg1:mCherry-F)* and *mpx:EGFP signals in* larvae infected E2Crimson- labeled *M. avium* or *M. marinum* as detailed in Fig. 6 (n=16 for *M. avium* infection, n=13 for *M. marinum* infection, N=2). Statistical significance of difference was determined by unpaired t-test. ns, not significant.


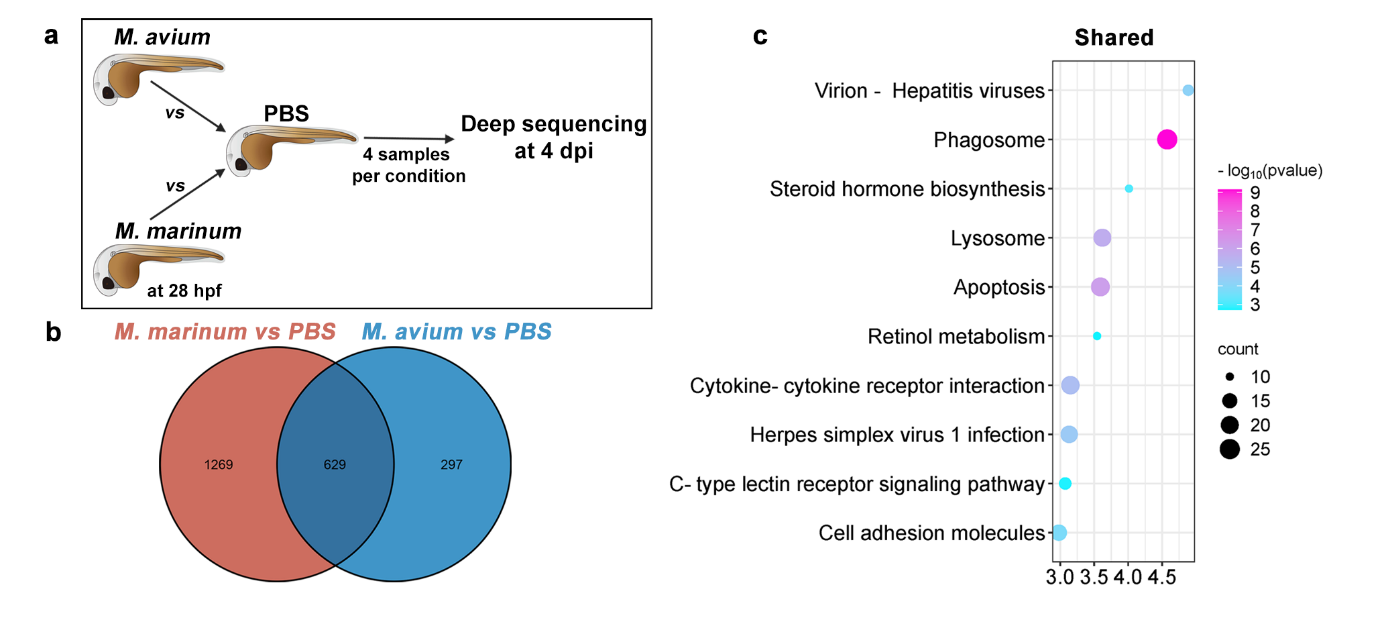
**Fig. S7 Experimental setup of transcriptomic analysis of *M. marinum* and *M. avium* infection. a** Experimental scheme of RNAseq samples collection as previously reported (23). *AB/TL* embryos were systematically infected with *M. avium*, *M. marinum* and PBS (control) at 28 hpf and larvae were collected for deep sequencing at 4 dpi. **b** The Venn diagram illustrates the number of shared and specific differentially expressed genes (DEGs) between *M. avium*- infected zebrafish larvae and *M. marinum*- infected zebrafish larvae in comparison to the PBS control. Genes with a FDR p-value < 0.05 were considered statistically significant. **c** Top 10 significantly enriched KEGG pathways from the shared DEGs in both *M .avium* infection group *vs* PBS control groups and *M. marinum* infection group *vs* PBS control groups. The KEGG enrichment analyses were performed by using DAVID. The size of the circle represents the number of enriched genes, with larger circles indicating a higher number of enriched genes in the pathway. The color of the circle corresponds to - log10 (P- value) and the fold change is indicated on the x- axis. See main Fig. 8 for the pathways specific for *M. avium* or *M. marinum.*

**Movie S1** Time-lapse imaging of *M. avium*-infected neutrophils are engulfed by macrophages.
**Movie S2** Time-lapse imaging of neutrophil reverse migration following *M. avium* infection in the tail fin.
**Movie S3** Macrophage tracking in *Tg(mpeg1:mCherry- F);TgBAC(mpx:EGFP)* larvae infected with *M. avium* in the tail fin.
**Movie S4** Neutrophil tracking in *Tg(mpeg1:mCherry- F);TgBAC(mpx:EGFP)* larvae infected with *M. avium* in the tail fin.
**Movie S5** 3D reconstruction of *Tg(mpeg1:mCherry- F);TgBAC(mpx:EGFP)* larvae systemically infected with *M. avium* at 4 dpi.
**Movie S6** 3D reconstruction of *Tg(mpeg1:mCherry- F);TgBAC(mpx:EGFP)* larvae systemically infected with *M. marinum* at 4 dpi.
